# Conjugated Lithocholic Acid Activates Hepatic TGR5 to Promote Lipotoxicity and MASLD‐MASH Transition by Disrupting Carnitine Biosynthesis

**DOI:** 10.1002/advs.202410602

**Published:** 2025-05-08

**Authors:** Senlin Lian, Meixi Lu, Luo Jiajing, Bin Zhang, Yi Fang, Xuran Wang, Minghua Zheng, Yan Ni, Guifang Xu, Yonglin Yang, Runqiu Jiang

**Affiliations:** ^1^ Department of Lab Medicine The First Affiliated Hospital of Anhui Medical University. MOE Innovation Center for Basic Research in Tumor Immunotherapy and Anhui Province Key Laboratory of Tumor Immune Microenvironment and Immunotherapy Hefei Anhui 230022 China; ^2^ Medical School of Nanjing University Nanjing Jiangsu Province 210993 China; ^3^ Department of Gastroenterology Affiliated Nanjing Drum Tower Hospital and Medical School of Nanjing University Nanjing Jiangsu Province 210008 China; ^4^ NAFLD Research Center Department of Hepatology the First Affiliated Hospital of Wenzhou Medical University Wenzhou 325035 China; ^5^ The Children's Hospital National Clinical Research Center for Child Health Zhejiang University School of Medicine Hangzhou 310052 China; ^6^ Department of Infectious Diseases The Affiliated Taizhou People's Hospital of Nanjing Medical University Taizhou 225300 China; ^7^ Department of Gastroenterology The First Affiliated Hospital of Anhui Medical University Hefei Anhui 230022 China

**Keywords:** carnitine, gamma‐butyrobetaine hydroxylase 1, *Gpbar1*, MASH, secondary bile acids, TRIM21

## Abstract

Conjugated lithocholic acid (LCA) plays a critical role in the development of metabolic dysfunction‐associated steatotic liver disease (MASLD). In this process, hepatocyte inflammation‐caused upregulation of its receptor, Takeda G protein‐coupled receptor 5 (TGR5) is a crucial factor. Serum bile acid profiling shows an increase in conjugated LCA, which correlates with disease severity. Depletion of *Gpbar1* in hepatocytes significantly protects against the progression from MASLD to metabolic dysfunction‐associated steatohepatitis (MASH) that is related to conjugated LCA. In vivo and in vitro experiments indicate that TGR5 activation in hepatocytes promotes lipotoxicity‐induced cell death and inflammation by suppressing de novo carnitine biosynthesis. Mechanistically, TGR5 binding to *CD36* facilitates E3 ubiquitin ligase *TRIM21* recruitment, leading to the degradation of BBOX1, a crucial enzyme in de novo carnitine biosynthesis. Targeting TGR5 therapeutically can restore carnitine biosynthesis, which may offer a potent strategy to prevent or reverse the transition from MASLD to MASH.

## Graphical abstract

Takeda G protein‐coupled receptor 5 (TGR5) serves as a central regulator in the metabolic dysfunction‐associated steatotic liver disease (MASLD)/metabolic dysfunction‐associated steatohepatitis (MASH) transition via conjugated lithocholic acid (LCA). Mechanistically, TGR5 orchestrates lipotoxicity‐induced cell death through its interaction with *CD36*, which facilitates *TRIM21* recruitment and BBOX1 degradation, ultimately disrupting de novo carnitine biosynthesis. Importantly, Targeting hepatic TGR5 prevents transition of MASLD/MASH.

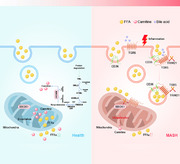

## 1 Introduction

Metabolic dysfunction‐associated steatotic liver disease (MASLD) encompasses a spectrum ranging from simple steatotic liver disease (SLD) to metabolic dysfunction‐associated steatohepatitis (MASH). It may involve fatty liver disease at various stages, potentially accompanied by hepatic fibrosis, cirrhosis, liver failure, and advanced hepatocellular carcinoma (HCC).^[^ ^1^, ^2^, ^3^ ^]^ The key distinction among these stages primarily depends on the presence of significant liver inflammation. In the initial stage, impaired lipid processing capability of the liver is the dominant pathological feature, which leads to substantial lipid accumulation in hepatocytes. Under this condition, hepatocytes may resist lipotoxic effects, and cell death remains relatively rare. However, as the disease progresses, various factors (including secondary insults) may overwhelm the ability of hepatocytes to manage lipid toxicity, thereby increasing immune‐related cell mortality and triggering inflammatory responses.^[^ ^4^, ^5^ ^]^ Inflammation may lead to progressive liver fibrosis and even cirrhosis in extreme cases, ultimately resulting in irreversible conditions (such as hypohepatia and liver cancer).^[^ ^6^ ^]^ Therefore, prevention of the transition from steatosis to MASH has become a primary focus in MASLD research.

Gastrointestinal (GI) metabolites play a crucial role in the transition from MASLD to MASH.^[^ ^7^ ^]^ These metabolites are predominantly produced by the gut microbiota and can affect hepatic metabolic pathways and immune responses, thus exacerbating liver inflammation and promoting the progression to more severe liver conditions.^[^ ^8^, ^9^ ^]^ An urgent but unsolved question is why GI metabolites that do not induce inflammation in healthy livers can promote the transition from MASLD to MASH. Our preliminary research has discovered that *CD36*, a free fatty acid (FFA) receptor on hepatocytes, can interact with the upregulated GI metabolite receptor TGR5 in an inflammatory environment. This interaction highlighted the critical role of receptor dynamics in the liver's response to GI metabolic signals.

Bile acids mediate their physiological and pathological effects through major cellular receptors, primarily the farnesoid X receptor (FXR) and TGR5.^[^ ^10^ ^]^ Compared with FXR, TGR5 exhibits a stronger response to GI metabolites, especially secondary bile acids (SBAs). TGR5 displays a more pronounced response to SBAs, particularly lithocholic acid (LCA) and its derivatives.^[^ ^11^ ^]^ There is a certain concentration of SBAs in healthy livers, but TGR5 is poorly expressed in healthy hepatocytes.^[^ ^12^ ^]^ The limited expression of TGR5 may help maintain the low reactivity of a healthy liver to GI metabolites. This suggests a protective mechanism against excessive inflammatory and metabolic responses under normal physiological conditions.

## 2 Results

### 2.1 TGR5 Reduces *CD36*‐Mediated Lipid Metabolism in Hepatocytes in an Inflammatory Microenvironment

**Figure 1:**
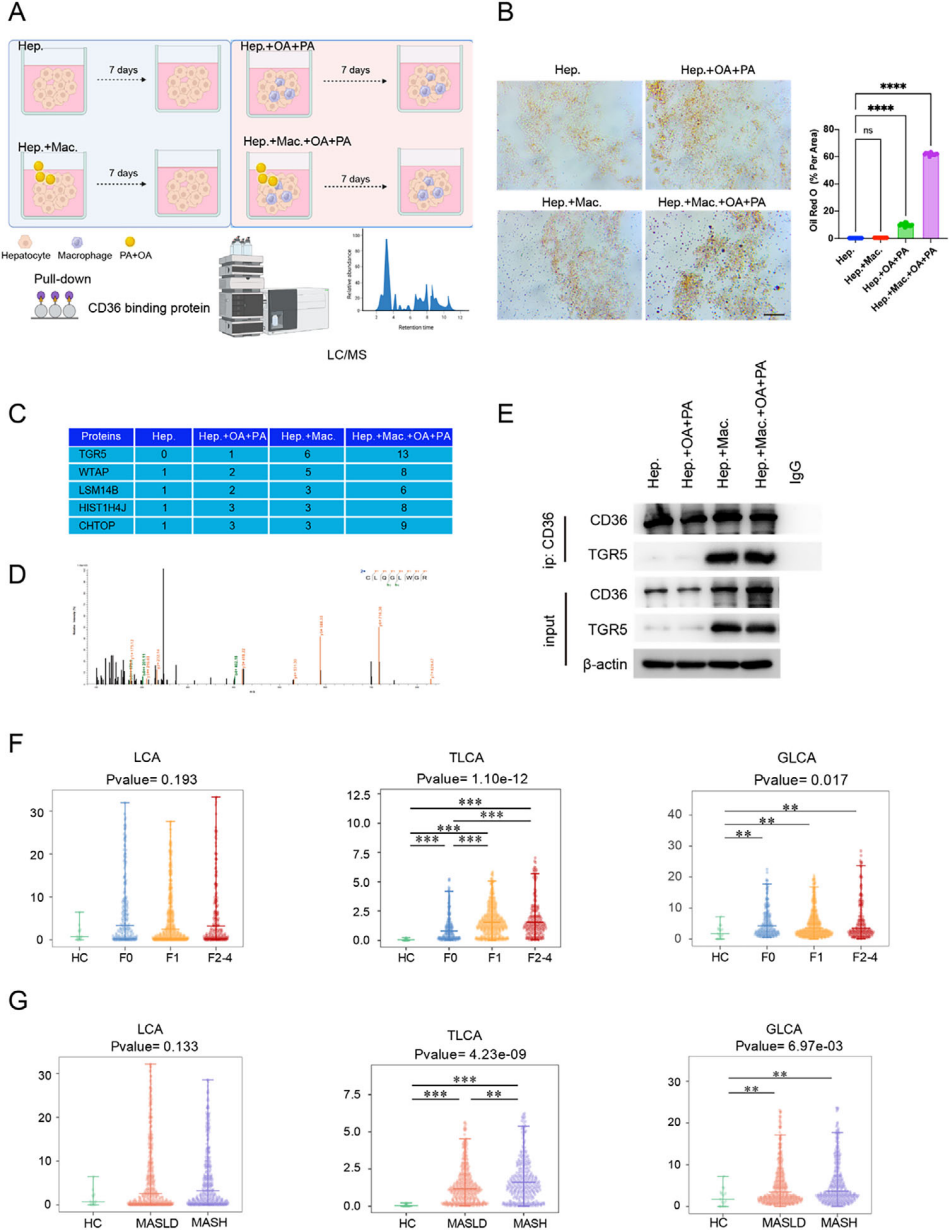
TGR5 reduces *CD36*‐mediated lipid metabolism in hepatocytes within an inflammatory environment. A) Schematic representation of the experimental setup for co‐culturing hepatocytes with macrophages and treating with oleic acid and palmitic acid (OA+PA). The pull‐down assay was performed to isolate *CD36* binding proteins, followed by LC/MS analysis to identify the protein interactions. B) Comparison of the effects of different treatment conditions on lipid accumulation in hepatocytes using Oil Red O staining. Statistical significance was determined using on one‐way ANOVA test with Dunnett adjustment, each group *n* = 6. C) LC/MS quantitative analysis of proteins (in peptides). D) Tandem mass spectrometry of TGR5. E) Co‐immunoprecipitation (Co‐IP) assay confirming the interaction between *CD36* and TGR5 in hepatocytes. F) Measurements of different serum bile acids in MASLD patients with different liver fibrosis stages. G) Comparing the serum concentration of different bile acids in patients with different MASLD stages (HC: healthy control, MASLD: metabolic associated steatohepatitis liver disease, MASH: metabolic associated steatohepatitis). Statistical significance was determined using one‐way ANOVA test with Dunnett adjustment. Data are presented as mean ± SD. All experiments were performed in triplicate. **p < 0.01, ***p < 0.0002, ****p < 0.0001, ns: *p* > 0.05.

The impact of inflammation on hepatocyte fatty acid metabolism was elucidated using a 3D culture model. Hepatocytes were isolated, co‐cultured with macrophages at a 1:1 ratio, and exposed to oleic acid (OA) and palmitic acid (PA) (**Figure** **1**A). As indicated by the results, hepatocytes co‐cultured with macrophages showed increased lipid droplet accumulation under exposure to OA+PA (Figure 1B), which suggested that macrophages inhibited hepatocyte lipid metabolism in an inflammatory environment. This may illustrate the immune‐metabolic interplay in hepatic tissues.

Given the critical role of *CD36* in hepatocyte FFA metabolism,^[^ ^13^ ^]^ this research investigated how inflammatory conditions might alter protein interactions with *CD36*. Specifically, *CD36*‐specific antibodies were used for immunoprecipitation to isolate *CD36*‐binding proteins under various conditions (Figure 1A). According to liquid chromatography‐mass spectrometry (LC/MS) results, TGR5 was identified as the most significantly upregulated protein in these complexes (Figure 1C,D). Co‐immunoprecipitation (Co‐IP) results confirmed the interaction between TGR5 and *CD36*. Notably, TGR5 was absent in hepatocytes under non‐inflammatory conditions. However, TGR5 was remarkably upregulated and bound to *CD36* after the introduction of OA and PA in hepatocyte‐macrophage co‐cultures (Figure 1E). The above findings suggested that TGR5 played a responsive role under lipid stress, indicating a dynamic regulatory mechanism in hepatic lipid metabolism and inflammatory response.

TGR5 is encoded by the *Gpbar1* gene and functions as a receptor for bile acids, especially GI metabolites SBAs.^[^ ^14^ ^]^ Consequently, the serum bile acids profiles, specifically LCA and its derivatives in various stages of liver fibrosis (F0–F4) in MASLD patients (*n* = 550) were investigated. It was found that as liver fibrosis or MASLD pathology progresses, the levels of LCA derivatives were notably increased, rather than LCA itself (Figure 1F,G).

### 2.2 TGR5 Expression Increases during the Transition from MASLD to MASH in an NF‐κB Signaling‐Dependent Manner

TGR5 expression dynamics in MASLD were investigated. In short, human liver samples were obtained and MASLD mouse models were constructed using high‐fat and high‐fructose (HFHfr) and Western diets (WD). Based on morphological assessments and serum hepatic enzyme levels, these samples were categorized into normal, MASLD, and MASH groups. Quantitative real‐time PCR (qRT‐PCR) and immunostaining were used to detect TGR5 expression in liver tissues at various MASLD stages. As revealed by the results, *Gpbar1* gene expression and its encoded protein TGR5 were significantly upregulated at the MASH stage (Figure S1A–C, Supporting Information). This suggested that increased TGR5 expression may be associated with the progression of MASLD to more severe stages.

Subsequently, the factors contributing to the transcriptional upregulation of the *Gpbar1* gene in hepatocytes were elucidated. The in‐vitro experiments were conducted to simulate three conditions that may influence hepatocytes in MASH, including supernatant from lipopolysaccharide (LPS)‐stimulated macrophages (SUP.), high‐fat (OA+PA), and high glucose (H.G.). The outcomes indicated that SUP. treatment notably elevated the transcription and protein levels of *Gpbar1* (Figure S1D,E, Supporting Information). In addition, *Gpbar1* expression in a transwell co‐culture system with macrophages and hepatocytes was determined. It was found that LPS‐stimulated macrophage secretion significantly enhanced *Gpbar1* gene expression, particularly at higher macrophage‐to‐hepatocyte ratios, suggesting a dose‐dependent relationship (Figure S1F, Supporting Information). Next, hepatocytes were exposed to TNFα, interleukin (IL)‐6, and IL‐1β to identify specific responsible macrophage cytokines. According to the results, only TNFα remarkably increased *Gpbar1* expression (Figure S1G, Supporting Information). Notably, TGR5 expression in hepatocytes was progressively increased when TNFα concentration exceeded 5 ng/mL (Figure S1H, Supporting Information). Furthermore, it was demonstrated that TNFα upregulated *Gpbar1* expression in hepatocytes via the NF‐κB signaling pathway (Figure S1I,J, Supporting Information). Taken together, the above results proposed a direct mechanistic link between TNFα signaling and the modulation of *Gpbar1* gene expression in hepatocytes.

Subsequently, the relationship between TGR5 and TNFα was analyzed in vivo in both human and mouse samples. Multiplex immunohistochemistry (mIHC) staining was performed to assess the presence of TGR5, TNFα, and CD68‐positive macrophages in MASLD tissues. The results revealed a significantly positive correlation between TGR5 and TNFα levels. In addition, there was a positive correlation between TGR5 expression and macrophage numbers in liver tissues (Figure S2A,B, Supporting Information). Further investigations revealed that higher TGR5 expression in hepatocytes was markedly associated with elevated levels of liver function markers (alanine aminotransferase [ALT], aspartate aminotransferase [AST], and alkaline phosphatase [ALP]) in MASLD patients (**Table** **1**). These findings suggested that TGR5 was a marker of inflammatory and metabolic disturbances in liver tissues and correlated with liver dysfunction severity in MASLD.

**Table 1:** Baseline characteristics of the clinical samples, according to the expression of TGR5^a^ ^)^

Variable	TGR5^high )^ (*N* = 44)	TGR5^low )^ (*N* = 45)	Total
**Demographic characteristics**			
Age (years)	56 ± 6	54 ± 8	56 ± 8
Sex‐no. (%)			
Male	20 (45)	23 (51)	43 (48)
Female	24 (55)	22 (49)	46 (52)
Body mass index^b^ ^)^			
Distribution‐no. (%)			
<25	2 (5)	1 (2)	3 (3)
25 to <30	7 (16)	15 (33)	22 (25)
30 to <35	13 (29)	17 (38)	30 (34)
≥35	22 (50)	12 (27)	34 (38)
**Histologic features**			
NAFLD activity score^c)^	5.2 ± 1.7	4.0 ± 1.5	4.2 ± 1.6
Steatosis score	1.6 ± 0.8	1.2 ± 0.6	1.5 ± 0.9
Lobular inflammation score	1.9 ± 0.7	1.4 ± 0.4	1.6 ± 0.8
Ballooning score	1.7 ± 0.6	0.8 ± 0.5	1.2 ± 0.6
Fibrosis stage^d^ ^)^	4.0 ± 0.0	0.9 ± 0.7	1.5 ± 1.2
MASH diagnosis‐no. (%)			
Definite or borderline MASH	41 (93)	27 (60)	68 (76)
MASLD, not MASH	3 (7)	18 (40)	21 (24)
**Liver tests**			
Median ALT(IQR) (U L^−1^)	40 (26–57)	48 (30–74)	52 (31–80)
Median AST(IQR) (U L^−1^)	45 (32–64)	33 (25–52)	36 (29–59)
Median ALP(IQR) (U L^−1^)	89 (73–114)	70 (56–87)	74 (62–92)

^a)^ ± values are means ± SD. Percentages may not total 100 because of rounding. IQR interquartile range, MASLD metabolic dysfunction‐associated steatotic liver disease, and MASH metabolic dysfunction‐associated steatohepatitis

^b)^ Body mass index is the weight in kilograms divided by the square of the height in meters. A body mass index of less than 25 indicates normal weight, of 25 to less than 30 overweight, 30 to less than 35 obese, and 35 or above morbid obesity.

^c)^ NAFLD activity score was assessed on a scale of 0–8, gauges disease severity through three criteria: steatosis (0–3 scale), lobular inflammation (0–3), and hepatocellular ballooning (0–2).

^d)^ Fibrosis stage was assessed on a scale of 0–4, with higher scores indicating more severe fibrosis.

### 2.3 Hepatocyte TGR5 Exacerbates Conjugated LCA‐Related MASLD Model

**Figure 2:**
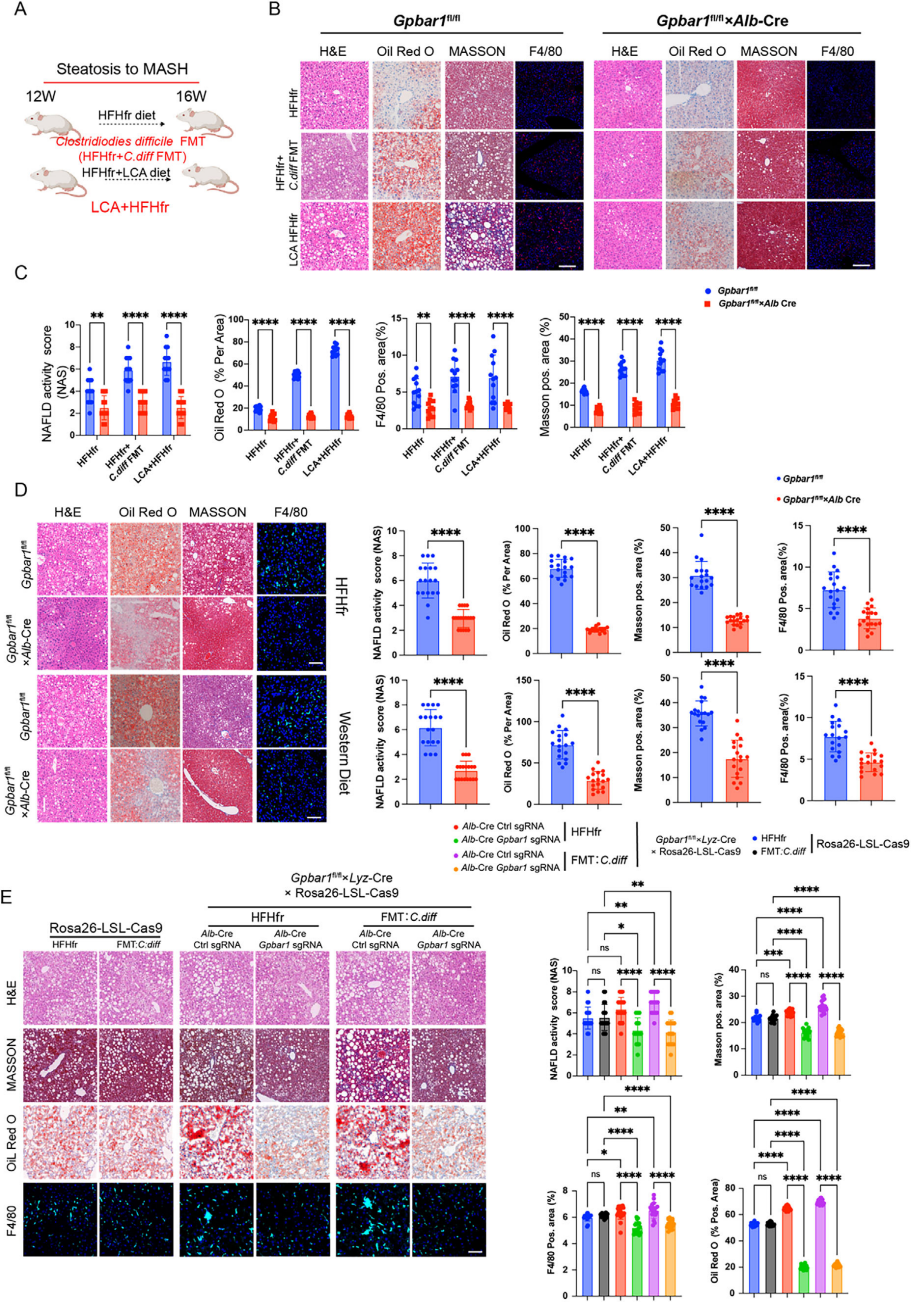
Hepatic TGR5 exacerbates conjugated LCA induced hepatic inflammation, fibrosis in in vivo mice models. A) A schematic representation of the construction of the two mouse MASH models. B,C) Comparison of H&E, Oil Red O, Masson trichrome, F4/80 immunofluorescence staining, and NAS scores in *Gpbar1* ^fl/fl^ and *Gpbar1* ^fl/fl^×*Alb*‐Cre mice at 16 weeks of the two conjugated LCA induced MASH model, each group *n* = 12. D) Comparison of H&E, Oil Red O, Masson trichrome, F4/80 immunofluorescence staining, and NAS scores in *Gpbar1* ^fl/fl^ and *Gpbar1* ^fl/fl^×*Alb*‐Cre mice at 20 weeks of HFHfr and WD diet MASH model, each group *n* = 18. E) Comparison of H&E, Oil Red O, Masson trichrome, F4/80 immunofluorescence staining, and NAS scores in Rosa26‐LSL‐Cas9 mice, *Gpbar1* ^fl/fl^×*Lyz*‐Cre cross with Rosa26‐LSL‐Cas9 mice with different treatment indicated in the figure until 16 weeks of HFHfr and HFHfr+FMT:*C. diff* mouse MASH model, each group *n* = 18. Data are presented as mean ± SD. All experiments were performed in triplicate. **p < 0.01, ***p < 0.0002, ****p < 0.0001, ns: *p* > 0.05.

Next, the impact of hepatic TGR5 expression in hepatocytes on MASLD was comprehensively investigated. Specifically, *Gpbar1* ^fl/fl^ mice were generated and crossed with *Alb*‐Cre mice for selective depletion of TGR5 expression in hepatocytes. Clinical bile acid profiling data have indicated that the natural TGR5 ligand glycine/taurine conjugated lithocholic acid (G/TLCA) significantly increases as MASLD progresses. Therefore, it is hypothesized that TGR5 may exacerbate hepatocyte damage. To verify this hypothesis, a series of experiments were conducted using two HFHfr mouse MASLD models: HFHfr + *Clostridium difficile* (*C. diff*) fecal microbiota transplantation (FMT) [treated with *C. diff* transplantation] and HFHfr + LCA (treated with a high LCA diet) (**Figure** **2**A). According to the results, both treatments remarkably exacerbated the MASH phenotype, with high LCA diet treatment showing a more pronounced effect. Interestingly, mice with hepatocyte‐specific depletion of *Gpbar1* did not show significant MASH progression, even with treatments of C. diff transplantation and a high LCA diet (Figure 2B,C and Figure S3A, Supporting Information). This suggested that TGR5 played a crucial role in mediating the liver's response to conjugated LCA, which can affect MASH development.

Next, the HFHfr and WD models at 8, 16, and 20 weeks were established to compare MASLD phenotypes at different time points. Similar to FMT and LCA diet mouse MASLD models, *Gpbar1* knockdown in hepatocytes noticeably inhibited MASLD progression in both models, as manifested as reduced hepatocyte vacuolization by H&E staining, decreased Oil Red O‐stained cells, attenuated hepatic fibrosis by Masson's trichrome staining, and alleviated hepatic inflammation by F4/80 staining, compared to *Gpbar1* ^fl/fl^ mice (Figure 2D and Figure S3B, Supporting Information). These differences were particularly prominent at 20 weeks (Figure 2D). Despite significant liver inflammation and fibrosis in *Gpbar1* ^fl/fl^ mice, hepatocyte‐specific *Gpbar1* knockout mice did not show these severe symptoms. In addition, hepatocyte‐specific *Gpbar1* knockout not only reduced mouse body weight but also improved serum markers. As indicated by serological analysis results, *Gpbar1* knockout mice exhibited markedly decreased ALT, TNFα, MCP1, TG, and TC levels compared to the control group (Figure S3C,D, Supporting Information).

Previous studies have shown that TGR5 is a protective factor in MASLD development in the liver, as TGR5‐knockout mice exhibit a more pronounced MASLD phenotype. Further research has indicated that TGR5 exerts an anti‐inflammatory effect in hepatic macrophages.^[^ ^15^, ^16^ ^]^ Hence, we then shifted to comparing the roles of macrophage and hepatic TGR5 in the progression of MASLD to MASH. In short, *Gpbar1* ^fl/fl^ mice were specifically knocked out of the *Gpbar1* gene in myeloid cells using *Lyz*‐Cre mice, with *Gpbar1* ^fl/fl^ mice as controls. The results in HFHfr and WD models demonstrated that TGR5 in myeloid cells can protect against MASLD onset and progression (Figure S3E, Supporting Information).

Furthermore, *Lyz*‐Cre‐Gpbar1fl/fl mice were crossed with Rosa26‐LSL‐Cas9 mice. At the 12th week of the HFHfr and *C. diff* FMT models, AAV8 carrying *Alb*‐Cre‐*Gpbar1* sgRNA was injected via the spleen for TGR5 knockout in hepatocytes. At the 16th week, it was observed that mice with hepatocyte TGR5 knockout showed significantly milder MASH phenotype compared to those with TGR5 knocked out in myeloid cells (Figure 2E). These results suggested that hepatocyte TGR5 played a dominant role in MASH progression.

### 2.4 TGR5 Facilitates Hepatocyte Death by Suppressing Hepatocyte Carnitine Synthesis

The above findings have pinpointed TGR5 as a pivotal factor exacerbating lipotoxicity and hepatocyte death within an in‐vivo MASLD model. To delve deeper into the underlying action mechanisms, we extended our research involving experiments using primary hepatocytes.

The impact of various factors on hepatocyte FFA processing was assessed using OA+PA treatment and Oil Red O staining. As revealed by the results, TNFα did not notably promote lipid accumulation, while OA+PA remarkably increased Oil Red O‐positive areas in wild‐type hepatocytes, not in *Gpbar1* knockout hepatocytes. TGR5 agonists (G/TLCA and INT‐777) further intensified FFA accumulation in AML12 cells (Figure S4A, Supporting Information). TNFα combined with TGR5 agonists elevated TG, TC, *Il6*, and *Il8* mRNA levels, particularly in wild‐type hepatocytes (Figure S4B, Supporting Information). As indicated by flow cytometry results, TNFα and TGR5 agonists significantly increased cell death in wild‐type hepatocytes, but not in *Gpbar1* knockout hepatocytes (Figure S4C, Supporting Information).

**Figure 3:**
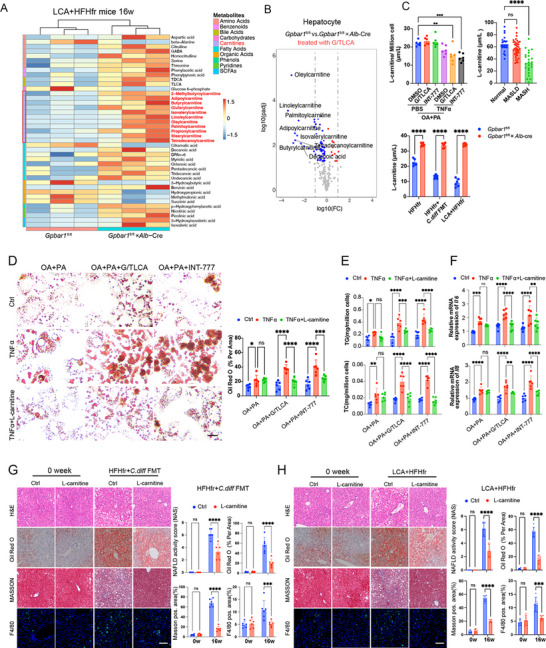
Hepatic TGR5 exacerbates conjugated LCA induced MASH by decreasing de novo carnitine synthesis. A) Heatmap of metabolomic profiling data showing differences in metabolite levels between *Gpbar1* ^fl/fl^ and *Gpbar1* ^fl/fl^×*Alb*‐Cre mice in LCA+HFHfr model at 16 weeks. B) Volcano plot comparing metabolites levels in hepatocyte isolated from *Gpbar1* ^fl/fl^ versus *Gpbar1* ^fl/fl^×*Alb*‐Cre mice and treated with 50 × 10^−6^ m G/TLCA. C) l‐carnitine concentration in hepatocytes treated differently, serum l‐carnitine levels in human liver samples (Normal *n* = 23, MASLD *n* = 39, MASH *n* = 24) and mouse (Gpbar1fl/fl vs *Gpbar1* ^fl/fl^×*Alb*‐Cre, each group *n* = 6) liver samples in two MASH model. Statistical significance was determined using one‐way ANOVA test with Dunnett adjustment. D) Representative figures and qualification of Oil Red O staining for mouse primary hepatocyte treated differently. Statistical significance was determined using two‐way ANOVA test with Dunnett adjustment, each group *n* = 6. E,F) Comparisons of TG, TC levels, and the transcription levels of the inflammatory cytokines *Il6* and *Il8* in primary mouse hepatocytes after treatment as shown in the figure. Statistical significance was determined using two‐way ANOVA test with Dunnett adjustment, each group *n* = 6. G,H) Comparison of Oil Red O, Masson's trichrome, and F4/80 immunofluorescence staining and NAS score in liver tissues obtained from two mouse MASH models treated as shown in the figure. Statistical significance of (H) and (I) was determined using one‐way ANOVA test with Šidák adjustment, each group *n* = 6. Data are presented as mean ± SD. All experiments were performed in triplicate. *p < 0.05, **p < 0.01, ***p < 0.0002, ****p < 0.0001, ns: *p* > 0.05.

RNA‐seq and metabolomics analyses on cells and mouse liver tissues were conducted to elucidate the potential mechanisms of hepatocytes in promoting MASLD progression. According to RNA‐seq analysis results of MASLD tissues from cells and LCA‐fed mice, differentially expressed genes were enriched in KEGG pathways related to cell death, fatty acid, and lipid metabolism (Figure S4D–F, Supporting Information). Notably, metabolomics assays demonstrated that wild‐type hepatocytes or Gpabr1fl/fl mouse liver tissues showed significantly decreased levels of carnitine metabolites compared to *Gpbar1* knockout hepatocytes treated with TNFα+OA+PA+INT‐777 or *Gpbar1* hepatocyte‐specific knock out MASLD mice treated with LCA diet (**Figure** **3**A,B, Supporting Information). We confirmed L‐carnitine concentration in cell lines, mouse MASH tissues, and human MASH samples (Figure 3C and Figure S4G, Supporting Information).

Subsequently, the role of reduced carnitine levels in the death of wild‐type hepatocytes was investigated following TNFα‐bound TGR5 agonist treatment. Specifically, 50 × 10^−6^ m l‐carnitine was added to wild‐type hepatocytes in vitro. This treatment reduced Oil Red O staining areas, cell death, intracellular TG and TC levels, and *Il6* and *Il8* mRNA levels (Figure 3D–F). In vivo, 0.28% l‐carnitine supplementation in wild‐type mouse from week 12 to 20 in HFHfr+*C. diff* FMT and HFHfr+LCA models effectively reduced nonalcoholic fatty liver disease activity score (NAS), Oil‐Red O staining areas, hepatic fibrosis (indicated by Masson's trichrome staining), cell death rate, and F4/80‐positive cells (Figure 3G,H).

### 2.5 TGR5 Decreases Hepatocyte De Novo Carnitine Biosynthesis by Degrading Gamma‐Butyrobetaine Hydroxylase 1 (BBOX1) Proteins

**Figure 4:**
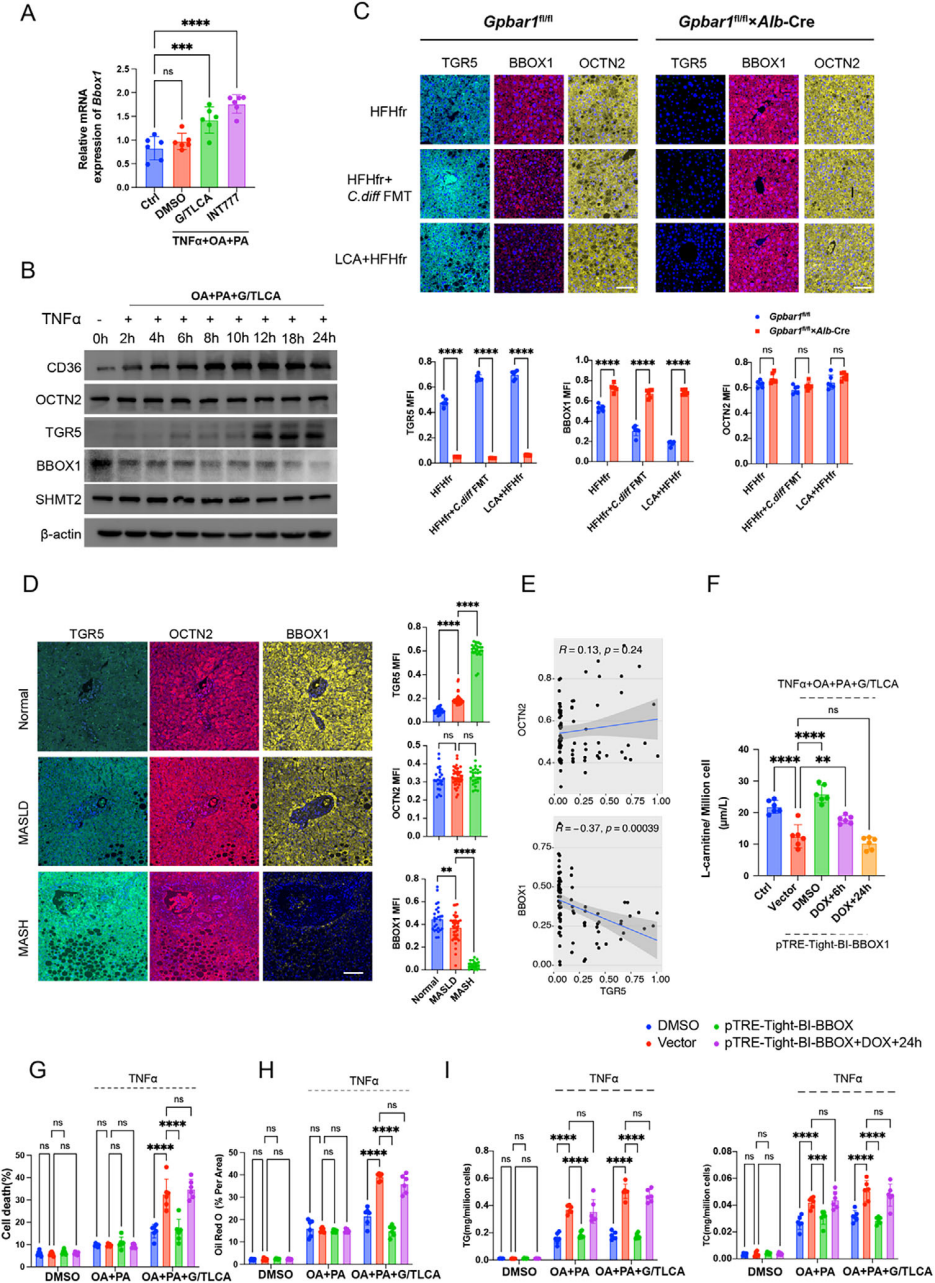
Activation of hepatic TGR5 decrease de novo carnitine biosynthesis by degrading BBOX1 proteins. A) Relative mRNA expression of *Bbox1* in hepatocytes with different treatment. Statistical significance was determined using one‐way ANOVA test with Dunnett adjustment, each group *n* = 6. B) WB monitors the protein expression of *CD36*, OCTN2, TGRR5, BBOX1, and SHMT2 in hepatocytes after the treatment shown in the figure, with β‐actin as the internal control. C) mIHC staining and mean fluorescence intensity (MFI) qualification of TGR5, BBOX, and OCTN2 expression in liver sections from *Gpbar1* ^fl/fl^ and *Gpbar1* ^fl/fl^×*Alb*‐Cre mice treated with two MSAH models. Statistical significance was determined using two‐way ANOVA test with Šidák adjustment, each group *n* = 6. D) mIHC staining and MFI qualification of TGR5, BBOX1, OCTN2 expression in human samples with normal liver *n* = 23, MASLD *n* = 39, and MASH *n* = 24. E) Correlation analysis of TGR5 with OCTN2 or BBOX1 expression in human liver samples. F) The l‐carnitine concentration in AML12 cells under different treatments, each group *n* = 6. G–I) Comparison of cell death rate, Oil Red O staining positivity, and intracellular TG and TC levels in AML12 cells under different transfections and treatments. Statistical significance was determined using two‐way ANOVA test with Dunnett adjustment, each group *n* = 6. Data are presented as mean ± SD. All experiments were performed in triplicate. **p < 0.01, ***p < 0.0002, ****p < 0.0001, ns: *p* > 0.5.

Research has indicated that TGR5 facilitates the transition from MASLD to MASH primarily by downregulating carnitine biosynthesis. This process involves the capacity of hepatocytes to synthesize carnitine from amino acids and uptake exogenous carnitine via the OCTN2 transporter. To investigate this mechanism, we evaluated the expression of relevant metabolic enzymes and transporters in cells and animals. As revealed by qRT‐PCR analysis results, the transcript levels of genes involved in carnitine synthesis (*Bbox1*, *Tmlhe*, *Aldh9a1*, and *Shmt2*) and carnitine transport (*Slc22a5*) remained largely unchanged with the combined action of TNFα, OA+PA, and the TGR5 agonist, except for *Bbox1* (**Figures** **4**A and S5A, Supporting Information). According to Western blot (WB) analysis results, OCTN2 (protein name of *Slc22a5*) and SHMT2 protein levels did not significantly change; however, BBOX1 level was almost completely degraded 24 h after treatment with TNFα, OA+PA, and the TGR5 agonist (Figure 4B). Furthermore, mIHC staining was used to evaluate the expression levels of BBOX1 and OCTN2. Notably, BBOX1 underwent significant degradation in models involving FMT with *C. difficile* and a high‐LCA diet, whereas OCTN2 remained unaffected. However, there were no significant changes in mice with hepatocyte‐specific depletion of *Gpbar1* (Figure 4C). Meanwhile, a significant decrease in BBOX1 protein level was observed at 20 weeks in both models, while OCTN2 expression level remained relatively stable over time (Figure S5B,C, Supporting Information). Moreover, similar results were observed in human MASLD tissues, where BBOX1 protein level was remarkably reduced in MASH patients’ tissues, while OCTN2 expression level remained relatively stable (Figure 4D). As revealed by the correlation analysis results, TGR5 expression was markedly associated with BBOX1 protein levels in human MASLD tissues (Figure 4E).

The above results from both in vivo and in vitro studies indicated that the BBOX1 protein level was remarkably decreased during MASLD progression, despite stable mRNA expression. To confirm this, a doxycycline (DOX)‐inducible system with the p‐TRE‐Tight‐BI plasmid was used, enabling controlled *Bbox1* overexpression, which can be suppressed by DOX. After DOX treatment, l‐carnitine levels were measured at 6 and 24 h. It was observed that l‐carnitine level returned to baseline after 24 h, similar to that in empty vector control cells in the presence of TNFα, OA+PA, and G/TLCA (Figure 4F). In addition, hepatocytes overexpressing BBOX1 showed similar outcomes to empty vector controls in terms of cell death rate, Oil Red O staining positivity, intracellular TG and TC levels, and *Il6* and *Il8* transcription after 24 h of DOX induction (Figure 4H,I and Figure S5D, Supporting Information). Notably, hepatocytes treated only with OA+PA and overexpressing BBOX1 showed notably less recovery compared to groups where TGR5 was activated by G/TLCA (Figure S5E, Supporting Information).

### 2.6 Hepatocyte TGR5‐*CD36* Interaction Facilitates *TRIM21* Recruitment and Downregulation of BBOX1

**Figure 5:**
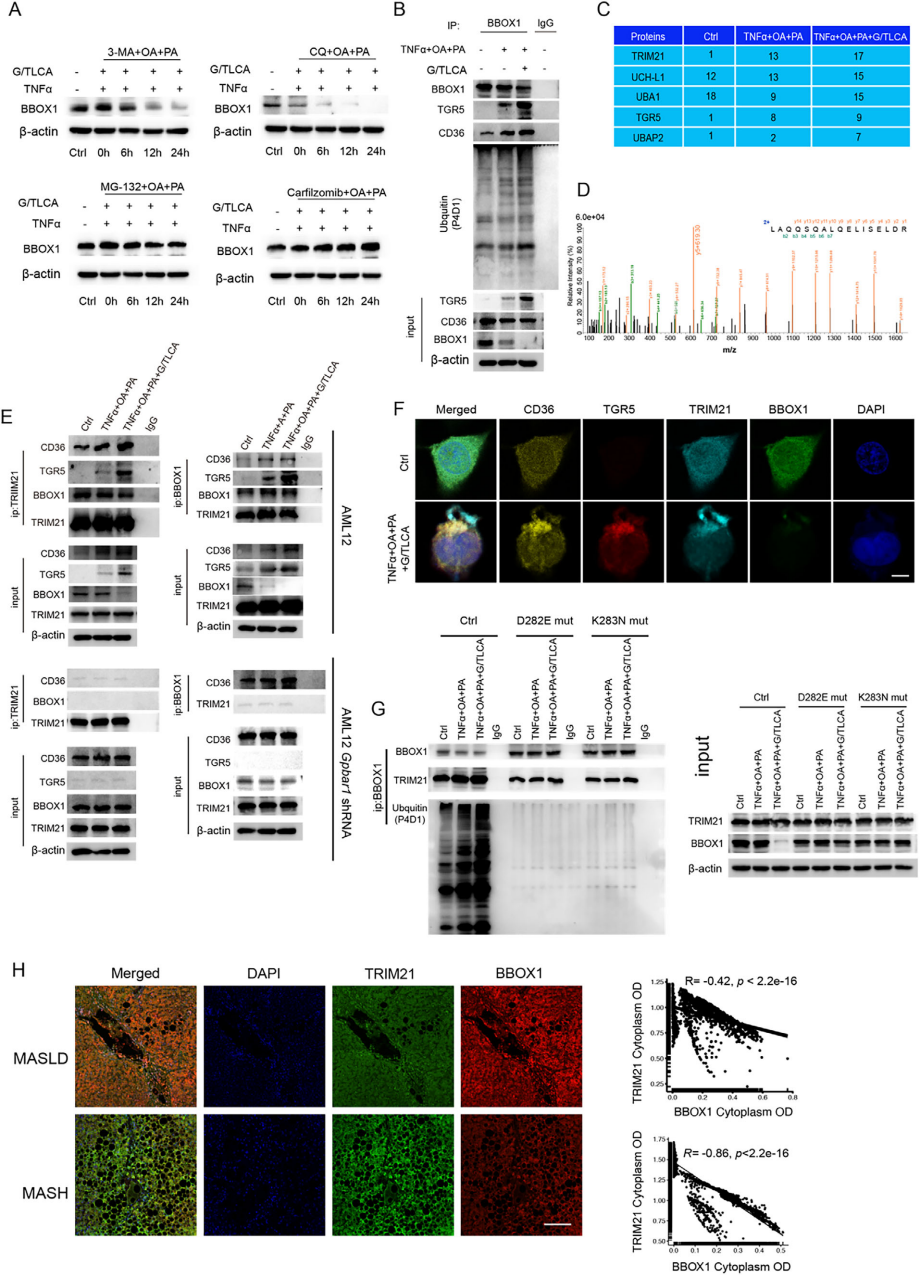
TGR5‐*CD36* interaction facilitates E3 ubiquitin ligase recruitment and downregulation of gamma‐butyrobetaine hydroxylase 1. A) Western blot analysis reveals the expression of BBOX1 in wild‐type hepatocytes at different time points upon treatment with autophagy inhibitors (3‐MA and CQ) and proteasome inhibitors (MG‐132 and Carfilzomib). B) Co‐immunoprecipitation (Co‐IP) and Western blot analysis of BBOX1 with ubiquitination, *CD36*, TGR5, and BBOX1 under treatment, with IgG as a negative control. C) The summarized table lists the top five proteins with the most significant differences identified in pull‐down LC/MS experiments using a BBOX1 antibody under different conditions. D) Tandem mass spectrometry of *TRIM21*. E) Co‐IP and WB verification show the interactions between *TRIM21*, *CD36*, TGR5, and BBOX1 in AML12 and AML12‐*Gpbar1* shRNA hepatocytes under different treatment conditions. IgG serves as a negative control. F) Co‐localization of TGR5 with *TRIM21* and *CD36* under different treatments by using laser confocal microscopy. G) Co‐IP and WB analyses reveal BBOX1 and its mutants (D282E, K283N) overexpression, BBOX1 ubiquitination, and the interaction between BBOX1 and *TRIM21* under different treatment conditions, with IgG serves as a negative control. H) mIHC is used to localize *TRIM21* and BBOX1 proteins within liver sections from MASLD and MASH patients. Correlation analysis indicates the relationship between OD values of *TRIM21* and BBOX1 in liver sections. Statistical significance of the correlations was determined using the Pearson correlation coefficient. Data are presented as mean ± SD. All experiments were performed in triplicate.

Subsequently, the mechanism of BBOX1 protein degradation in response to TGR5 activation was investigated. Specifically, hepatocytes were treated with autophagy inhibitors (3‐methyladenine [3‐MA] and chloroquine [CQ]) and proteasome inhibitors (MG‐132 and Carfilzomib). The results indicated that only the proteasome inhibitors effectively blocked BBOX1 protein degradation (**Figure** **5**A). According to ubiquitination level detection results, treatment with TNFα+OA+PA+G/TLCA notably increased BBOX1 protein ubiquitination, especially with the TGR5 agonist G/TLCA (Figure 5B). Next, the E3 ubiquitin ligase responsible for BBOX1 degradation was detected. Pull‐down studies were conducted using a BBOX1 antibody under 3 conditions: control, TNFα+OA+PA, and TNFα+OA+PA+G/TLCA. According to LC/MS and GO enrichment analysis results, the identified proteins were enriched in ubiquitination pathways (Figure 5C,D and Figure S6A, Supporting Information). Our analysis results revealed that the binding of the E3 ubiquitin ligase *TRIM21* increased progressively with TNFα+OA+PA and G/TLCA treatment.

Next, the interaction between BBOX1 protein and *TRIM21* was validated through Co‐IP and mIHC staining experiments. As revealed by these experiment results, the interaction between BBOX1 and *TRIM21* was enhanced with increasing TGR5 expression and activation (Figure 5E,F). Notably, in *Gpbar1* or *Cd36* knockdown hepatocytes, there was no binding between the BBOX1 protein and *TRIM21* (Figure 5E and Figure S6B, Supporting Information). This indicated the crucial role of TGR5‐*CD36* interaction for the binding of BBOX1 and *TRIM21*.

Subsequently, the mechanism underlying *TRIM21*‐mediated ubiquitination and degradation of BBOX1 was elucidated. The interaction sites between the proteins were predicted using ROSETTA software. Our analysis results indicated that *TRIM21* bound to BBOX1 at an aspartic acid residue at position 282 (Figure S6C, Supporting Information). A potential E3 ubiquitination site at lysine 283^[^ ^17^ ^]^ was identified, which was adjacent to the aspartic acid at position 282. We hypothesized that BBOX1's interaction with *TRIM21* at these residues may facilitate ubiquitination. For verification of this hypothesis, BBOX1 mutants D282E and K283N were generated and Co‐IP experiments were conducted. The results showed that both mutants reduced BBOX1 ubiquitination and degradation (Figure 5G,H, Supporting Information). AML12 overexpressing these mutants maintained lipid metabolism even with TNFα+OA+PA+G/TLCA treatment (Figure S6D, Supporting Information) and exhibited reduced cell death compared to wild‐type cells (Figure S6E, Supporting Information). In addition, TG and TC levels were markedly decreased (Figure S6F, Supporting Information). mIHC analysis of human MASLD samples demonstrated a linear negative correlation between *TRIM21* and BBOX1 expression at the MASH stage (Figure 5H). From the above compelling findings, it can concluded that TGR5, when activated in hepatocytes as a bridge docking *CD36*, promoted the E3 ubiquitination ligase *TRIM21* to catalyze the ubiquitination of BBOX1, which in turn degraded BBOX1.

### 2.7 Inhibiting TGR5 Activity In Vitro Prevents and Reverses the Progression of Conjugated LCA‐Induced Steatosis to MASH

**Figure 6:**
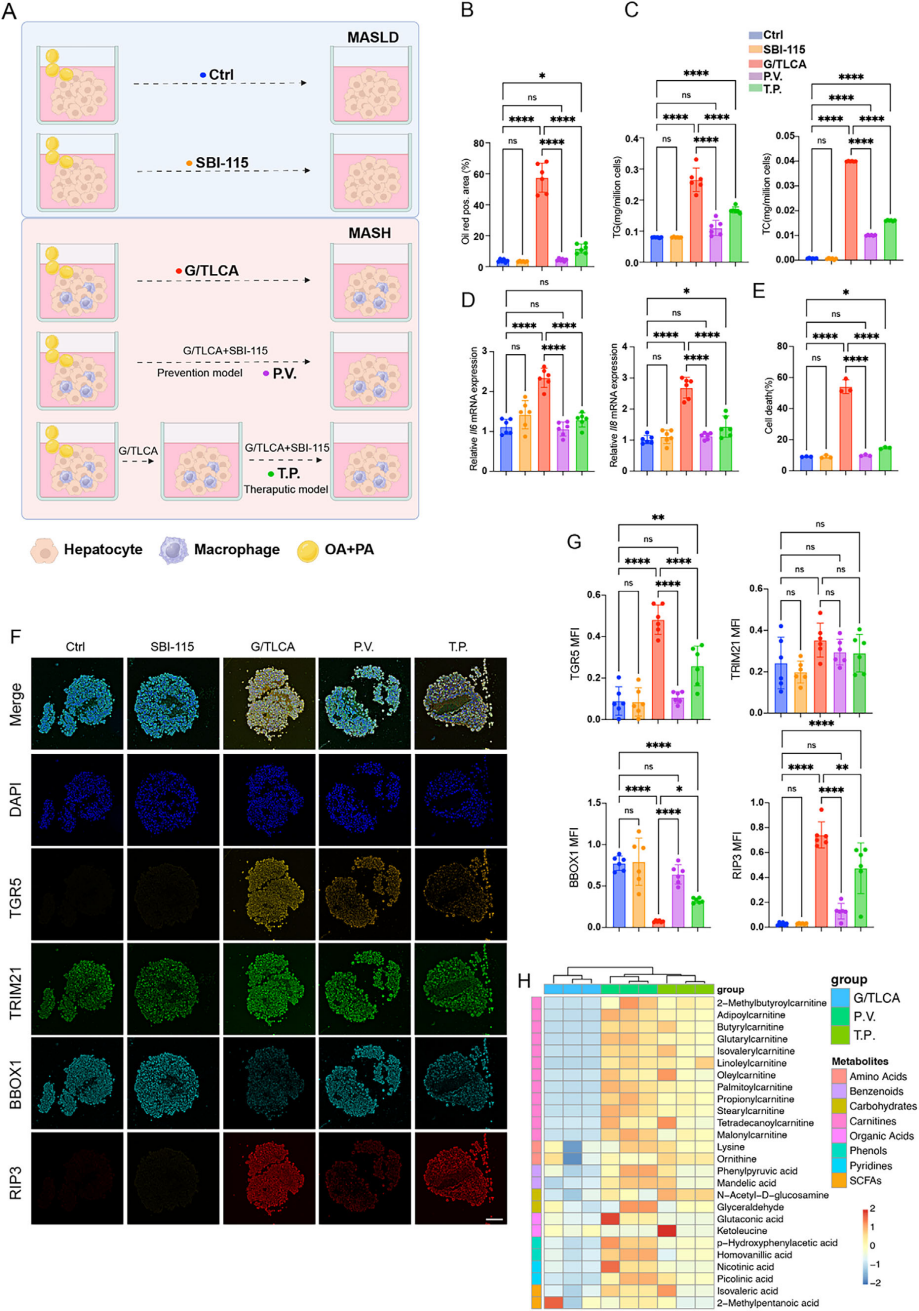
Inhibiting TGR5 activity has the potential to prevent and reverse the progression of MASLD to MASH in vitro. A) A schematic diagram illustrating how 3D culture system models conditions. B–E) Comparison of the Oil Red O staining positive area, TG and TC levels, transcription of *Il6* and *Il8* and cell death rate under different treatments in the in vitro MASH model, each group *n* = 6. F) Representative images showing the expression of TGR5, *TRIM21*, BBOX1, and RIP3 under different treatments in the in vitro MASH model using mIHC staining. G) Comparison of the MFI of TGR5, *TRIM21*, BBOX1, and RIP3 in the in vitro MASH model under different treatments. Statistical significance was determined using on one‐way ANOVA test with Šidák adjustment, each group *n* = 6. H) Heatmap showing metabolites levels under different treatments. Data are presented as mean ± SD. All experiments were performed in triplicate. *p < 0.05, **p < 0.01, ***p < 0.0002, ****p < 0.0001, ns: *p* > 0.05.

For investigating whether targeting hepatocyte TGR5 and its downstream pathways can halt the progression from MASLD to MASH, an in‐vitro model was established using 3D culture techniques, including two systems: primary hepatocytes alone and a co‐culture with macrophages. Two treatment scenarios were tested: inducing MASH with G/TLCA and using SBI‐115 (a TGR5 receptor inhibitor), either as a “prevention model” (administered simultaneously with G/TLCA) or a “therapeutic model” (introduced 48 hours after G/TLCA treatment) (**Figure** **6**A).

Initially, SBI‐115 did not enhance lipid metabolism in the isolated primary hepatocytes, as indicated by Oil Red O staining findings, as well as detection results of cell viability, and TG and TC levels. However, in hepatocytes co‐cultured with macrophages, SBI‐115 significantly reduced Oil Red O staining positive area, improved cell viability, and decreased TG and TC levels in the prevention model. It also reduced the production of inflammatory factors (such as *Il8* and *Il6*) within the 3D culture system. Remarkably, even with the occurrence of the transition from steatosis to MASH, SBI‐115 treatment can reverse this transition, exhibiting therapeutic effects similar to the prevention model (Figure 6B–E and Figure S7A,B, Supporting Information). These findings suggested that targeting TGR5 and its downstream pathways with SBI‐115 could effectively prevent and treat the progression from simple fatty liver to steatohepatitis, even after the disease has developed.

The therapeutic impact of SBI‐115 was further elucidated using mIHC staining and metabolomics. In the steatosis model, SBI‐115 treatment did not significantly alter TGR5 expression, *BBOX1* protein level, or hepatocyte cell death rate (as indicated by RIP3 positivity). However, in both the therapeutic and prevention models, SBI‐115 treatment notably preserved BBOX1 protein level and reduced cell death rates. (Figure 6F,G and Figure S7B,C, Supporting Information). Metabolomics analysis further confirmed these findings, showing that the inhibition of downstream TGR5 activity by SBI‐115 effectively restored carnitine metabolite levels in hepatocytes (Figure 6H).

### 2.8 Targeting TGR5 and Its Downstream Pathways Is Effective for Reversing Conjugated LCA‐Induced MASLD to MASH Progression In Vivo

**Figure 7:**
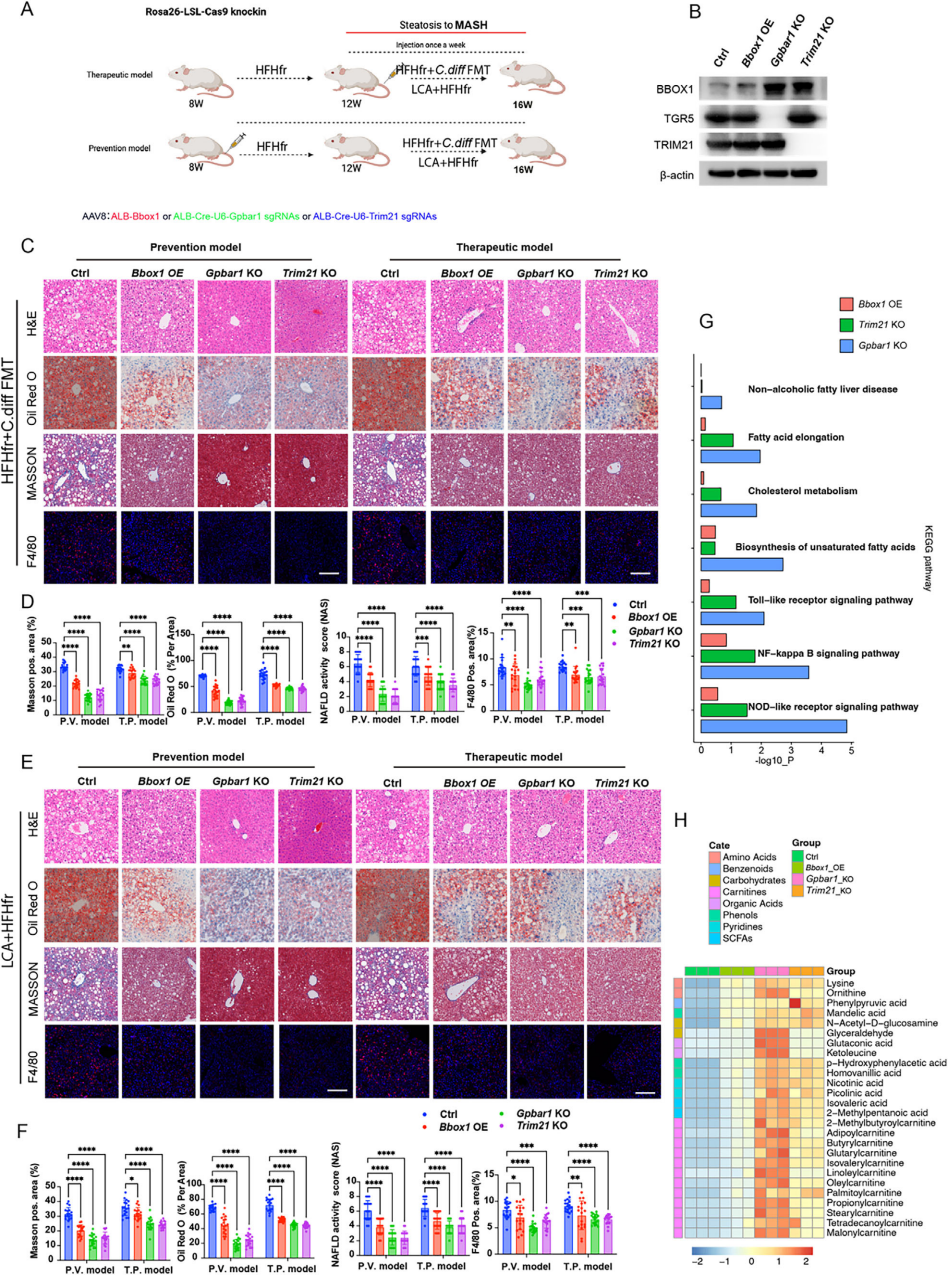
In vivo therapies targeting hepatocyte TGR5 and downstream pathways effectively reverse the progression from LCA‐induced MASLD to MASH. A) Experimental design illustrating the therapeutic and prevention models for the progression from conjugated LCA induced steatosis to MASH. B) WB analysis of BBOX1, TGR5, and *TRIM21*, with β‐actin expression in hepatocytes of mice treated indicated in the figure. C) Representative images of H&E, Oil Red O, Masson trichrome, and F4/80 immunofluorescence staining reflect the effects of prevention and therapeutic treatment on mice in different groups in the HFHfr+*C. diff* FMT mouse MASH model. D) Quantitative analysis of H&E, Oil Red O, Masson trichrome, and F4/80 immunofluorescence staining in the liver of mice in the HFHfr+*C. diff* FMT mouse MASH model with prevention and therapeutic treatment. Statistical significance was determined using on two‐way ANOVA test with Dunnett adjustment, each group *n* = 18. E) Representative images of H&E, Oil Red O, Masson trichrome, and F4/80 immunofluorescence staining reflect the effects of prevention and therapeutic treatment on mice belong to different groups in the LCA+HFHfr mouse MASH model. F) Quantitative analysis of H&E, Oil Red O, Masson trichrome, and F4/80 immunofluorescence staining in the liver of mice in the LCA+HFHfr mouse MASH model with prevention and therapeutic treatment. Statistical significance was determined using two‐way ANOVA test with Dunnett adjustment, each group *n* = 18. G) GSEA analysis based on the KEGG dataset reflects the impact of treatment and preventive treatment on genes related to lipid metabolism, inflammation, and fibrosis in mice. H) The heatmap shows the levels of four groups of indicated metabolites in the therapeutic model. Data are presented as mean ± SD. All experiments were performed in triplicate. *p < 0.05, **p < 0.01, ***p < 0.0002, ****p < 0.0001.

Considering the promising results of in vitro inhibition of TGR5 activity, its efficacy in vivo was evaluated. Currently, the specific inhibitors for hepatocyte‐targeted TGR5 have not been developed. To address this, Rosa26‐LSL‐Cas9 knock‐in mice were used with two mouse models of conjugated LCA‐induced MASLD and AAV8 virus‐mediated gene therapy was administered via the tail vein (**Figure** **7**A). The roles of manipulating hepatocyte TGR5 and *TRIM21*, as well as overexpressing BBOX1 and other therapeutic targets, in the progression from steatosis to MASH were explored in this study, considering both “therapeutic” and “prevention” approaches (Figure 7A).

Initially, the accurate expression of target proteins in isolated hepatocytes in vitro was confirmed using WB (Figure 7B). Next, the effects of the “therapeutic” and “prevention” models in mouse MASH models were assessed. In both models, the three treatments were effective when administered either early (at 8 weeks) or starting from the 12th week. Compared to the control group, overexpression of BBOX1 in hepatocytes or specific knockdown of *Gpbar1* and *Trim21* genes reduced liver vacuolation and the proportion of Oil Red O positive staining areas. Similarly, promising results were also observed in assessments of liver fibrosis by Masson's trichrome staining and liver inflammation by F4/80 staining (Figure 7C–F). Notably, simultaneous targeting of these three factors (particularly knockdown of *Gpbar1* in hepatocytes) significantly reduced mouse body weight. Serological tests confirmed the effectiveness of these treatments, as evidenced by protected liver function and reduced serum levels of TG, TC, and inflammatory factors (MCP1 and TNFα) (Figure S8A,B, Supporting Information).

Moreover, RNA‐seq analysis was used to assess the therapeutic effects. The results revealed that hepatic lipid metabolism capacity was substantially enhanced, accompanied by reduced inflammation and fibrosis levels, and post‐targeted prevention or treatment reflected by GSEA analysis (Figure 7G). Metabolomics analysis results further supportably indicated that hepatic carnitine metabolite levels were markedly improved (Figure 7H).

## 3 Discussion

It has been shown that persistent inflammation plays a pivotal role in the progression of steatosis to steatohepatitis.^[^ ^18^ ^]^ Inflammation arises from hepatocyte death, triggered by excessive accumulation of lipids, ultimately resulting in lipotoxicity and cell death.^[^ ^19^ ^]^ A previous study has revealed that hepatocyte lipid metabolism is crucial for counteracting lipotoxicity‐induced cell death, and *CD36* acts as a vital transporter in hepatocyte lipid metabolism.^[^ ^13^ ^]^ Hence, in this study, an in‐vitro 3D culture experiment was conducted to investigate whether inflammation can affect the functionality of *CD36*. Consequently, our research proved consistent results with previous studies, revealing that inflammation increased lipid accumulation in hepatocytes; LC‐MS analysis identified TGR5 as the most significantly increased protein in its binding to *CD36*, compared to the non‐inflammatory environment.

This discovery is of significant importance as *CD36* combined to TGR5 is associated with a substantial increase in hepatocyte mortality, primarily due to severe impairment of *CD36* and its normal function. *CD36*, also known as fatty acid translocase (FAT), is a cell surface protein that plays a pivotal role in the metabolism of FFAs in various tissues.^[^ ^20^, ^21^, ^22^ ^]^ It has been shown that *CD36* can actively participate in hepatocyte lipid metabolism through diverse functions such as FFA uptake, fatty acid storage, energy production, insulin sensitivity, etc.^[^ ^23^, ^24^, ^25^ ^]^ The function of *CD36* depends on a fundamental cellular process called endocytosis, which is a vital means for cells to transport extracellular biomacromolecules.^[^ ^26^ ^]^ Given the binding between TGR5 and *CD36*, both membrane receptors are present within entotic vesicles. Our discovery indicated that TGR5 can serve as a bridge to facilitate the degradation of BBOX1, a pivotal protein responsible for FFAs entering mitochondria through the ubiquitination proteasome pathway. Consequently, this process hinders the beta‐oxidation of FFAs before vesicles enter the mitochondria.

TGR5, short for G protein‐coupled bile acid receptor 1 (*Gpbar1*), is a cell membrane receptor that can significantly regulate various physiological processes.^[^ ^14^, ^27^, ^28^ ^]^ TGR5 primarily responds to bile acids and participates in controlling metabolism, inflammation, and energy balance.^[^ ^12^, ^14^ ^]^ It is present in many tissues throughout the body, including the liver, intestine, and immune cells.^[^ ^14^ ^]^ A previous study has pointed out that TGR5 exhibits a higher sensitivity to SBAs than to primary bile acids.^[^ ^29^ ^]^ Notably, the existing research has indicated that TGR5 expression is absent in healthy hepatocytes,^[^ ^30^, ^31^ ^]^ which serves as a protective mechanism against the hepatic effects of bile acid originating from intestinal bacteria.^[^ ^32^ ^]^ Our investigation revealed that, in the presence of inflammatory stimulation, the *Gpbar1* gene was transcriptionally active under the regulation of NF‐κB. This elevated expression enhanced the responsiveness of both murine and human liver cells to SBAs. Furthermore, our data elucidated that a notable elevation in serum concentrations of SBAs (particularly LCA and its derivatives) was correlated with MASH fibrosis progression. These data strongly supported that TGR5 may serve as a promising target for MASH prevention or treatment.

We found that TGR5 in hepatocytes emerges in an inflammatory environment and can promote MASH development by inducing hepatocyte death. Nonetheless, TGR5 is a classic metabolism‐related receptor that plays a relatively well‐understood role in macrophages, especially hepatic macrophages, where it exerts an anti‐inflammatory effect by inhibiting the NF‐κB pathway activation.^[^ ^15^, ^16^ ^]^ Therefore, in the MASLD model of macrophage TGR5 knockout mice, TGR5 plays a protective role, which is opposite to its role in hepatocytes. To address this issue, we used a dual knockout model and found that when TGR5 in hepatocytes was further knocked out at 16 weeks in the MASH model, the MASH phenotype in the dual knockout mice was noticeably alleviated, whether compared to macrophage TGR5 knockout mice or wild‐type (WT) mice. These results indicated that TGR5 on hepatocytes, rather than on macrophages, plays a more important role in the progression of MASH. The reason for this phenomenon may be that TGR5 inhibits the activation of the NF‐κB pathway. In hepatocytes, the NF‐κB pathway activation is generally considered protective, especially during inflammatory injury, as it promotes the expression of anti‐apoptotic genes and helps maintain liver function. However, in macrophages, the NF‐κB pathway activation is primarily associated with the cell's ability to promote inflammation.^[^ ^33^ ^]^ Under non‐inflammatory conditions, hepatocytes do not express TGR5. However, once TGR5 expression is upregulated, the de novo synthesis of carnitine‐related substances is reduced, resulting in a significant decrease in beta‐oxidation in hepatocytes, accompanied by a marked increase in hepatocyte death. This phenomenon may also be attributed to the inactivation of the NF‐κB pathway caused by elevated TGR5 levels in hepatocytes. Therefore, although the activation of TGR5 and its downstream effects in macrophages are associated with reduced pro‐inflammatory capacity, its activation in hepatocytes primarily leads to cell death. Both phenomena may be linked to the NF‐κB signaling pathway, highlighting the cell type‐specific roles of TGR5.

TGR5 inhibitors and CRISPR/Cas9 techniques were used for both in vivo and in vitro targeted interventions, yielding favorable outcomes, as shown by improved liver fibrosis, mitigated liver inflammation, and augmented hepatic lipid metabolism. It is worth noting that inhibition of TGR5 receptor activity has emerged as an efficacious therapeutic strategy. The primary mechanism underlying this intervention involves the disruption of *TRIM21* recruitment around the BBOX1 protein when TGR5 activity is inhibited or knocked out. Consequently, this intervention could impede the efficient degradation of BBOX1 protein, thereby safeguarding the lipid metabolic capacity of hepatocytes, alleviating the burden of lipid‐induced cytotoxicity, and ultimately leading to reduced hepatocyte mortality.

Carnitine is a natural compound in various foods, especially in meat and dairy products, which plays a crucial role in the metabolism of fatty acids. The primary function of carnitine is to transport long‐chain fatty acids to the mitochondria, the energy‐producing organelles within cells. Carnitine facilitates the movement of these fatty acids across the mitochondrial membranes, where they undergo beta‐oxidation, a process in which fatty acids are broken down to produce ATP.^[^ ^34^ ^]^ In addition, carnitine can be synthesized de novo within the human body from amino acids, primarily in the liver and kidneys, using specific amino acids and cofactors.^[^ ^35^ ^]^ Our in vivo investigations demonstrated that the administration of exogenous carnitine could effectively prevent MASLD development. Nonetheless, it is noteworthy that exogenous supplementation alone cannot rectify the diminished capacity of hepatic cells to biosynthesize carnitine. This limitation is associated with the interaction between TGR5 and the E3 ubiquitination ligase *TRIM21*, which leads to the degradation of BBOX1, a pivotal enzyme in the de novo carnitine biosynthesis pathway, specifically via the K283 site.

*TRIM21* is a member of the TRIM family of proteins and has gained much attention for its multifaceted roles in immune regulation, intracellular signaling, and protein degradation.^[^ ^36^ ^]^ In the context of MASH, the involvement of *TRIM21* is particularly intriguing. Recent research has shed light on the potential effect of *TRIM21* on MASH pathogenesis. It is believed that *TRIM21* can modulate the immune response and inflammation within the liver and influence the severity of liver injury and fibrosis. In addition, *TRIM21* may play a role in the regulation of cellular responses to oxidative stress, which is a key contributor to MASH development and progression.^[^ ^37^, ^38^ ^]^ Furthermore, it has been shown that the capacity of *TRIM21* to target specific proteins for ubiquitination and subsequent degradation is crucial in MASH.^[^ ^39^ ^]^ Taken together, *TRIM21* may affect the degradation of proteins involved in lipid metabolism, inflammation, and fibrosis, thereby affecting the overall course of the disease.

In summary, our investigation initially focused on the influence of inflammation on *CD36*‐mediated lipid metabolism. Remarkably, we uncovered a novel phenomenon that TGR5, a *CD36*‐bound SBA receptor, was expressed on hepatocytes in response to inflammatory cues. This unregulated TGR5 expression, when overexpressed, disrupted the crucial supply route of FFAs into mitochondria. This disruption was achieved through recruiting E3 ubiquitination enzyme *TRIM21*, leading to the degradation of BBOX1, an enzyme pivotal in the rate‐limiting step of the de novo carnitine biosynthesis pathway. Consequently, this disruption amplified lipid accumulation within hepatocytes and intensified lipotoxicity, ultimately triggering cell death, inflammation, and eventually liver fibrosis. In this study, critical factors (including TGR5, carnitine metabolism, and *TRIM21*) were pinpointed as potential targets for preventing and treating the transition from simple fatty liver to MASH. These findings not only offer novel therapeutic approaches for this condition but also significantly enhance our understanding of the underlying mechanisms.

## 4 Experimental Section

### Human Study

Participants for this study were enrolled between January 2021 and June 2022 at the liver clinic associated with the First Affiliated Hospital of Anhui Medical University, Gulou Hospital, and People's Hospital of Taizhou. Inclusion criteria were adults between the ages of 35 and 65 years, and all participants were categorized as Healthy control, MASLD, and MASH, as evidenced by histopathological analysis of liver biopsy specimens. Liver histopathology for all study groups was independently verified by a pair of medical society‐certified pathologists to ensure the validity of inclusion. Individuals with one of the following conditions were excluded from participation in this study: documented history of chronic viral hepatitis, excessive alcohol consumption, or other diagnoses of liver disease. Liver biopsy samples were collected using an ultrasound‐guided technique. A venous blood sample was also collected from each participant for further analysis. All collected samples were rapidly frozen and preserved in liquid nitrogen and then stored at −80 °C until analysis. The study was approved by the Institutional Ethics Committee of the First Affiliated Hospital of Anhui Medical University to ensure compliance with ethical standards and participant safety.

Cohort from Wenzhou is a well‐characterized Prospective Epidemic Research Specifically of MASH (PERSONS) cohort. All MASLD patients in this study were consecutively recruited from 2016 to 2019 at the First Affiliated Hospital of Wenzhou Medical University in Wenzhou (China). The inclusion and exclusion criteria and liver histology assessment have been described extensively elsewhere.^[^ ^1^ ^]^ Written informed consent was obtained from each subject before study participation. The research protocol was approved by the ethics committee of the First Affiliated Hospital of Wenzhou Medical University (2016‐246, December 01, 2016) and registered in the Chinese Clinical Trial Registry (ChiCTR‐EOC‐17013562). A total of 550 Chinese adults with biopsy‐proven MASLD and 15 heathy volunteers as controls were included in the present study. Serum samples were collected for targeted metabolomics analysis of bile acids that had been reported in a previous work.^[^ ^2^ ^]^

### Mouse Models

C57BL/6 mice, *Gpbar1* ^fl/fl^ mice, *Alb‐*Cre mice, and *Lyz*‐Cre mice were purchased from GemPharmatech, while Rosa26‐LSL‐Cas9 knockin mice were obtained from The Jackson Laboratory. To generate hepatocyte‐specific deletions of *Gpbar1*, *Gpbar*1^fl/fl^ mice were crossed with *Alb*‐Cre mice. To generate myeloid‐specific deletions of *Gpbar1*, *Gpbar*1^fl/fl^ mice were crossed with *Lyz*‐Cre mice. To generate myeloid‐specific *Gpbar1* knockout mice with Cas9, we crossed myeloid‐specific *Gpbar1* knockout mice (*Gpbar1* ^fl/fl^ × *Lyz*‐Cre) with Rosa26‐LSL‐Cas9 knockin mice. All procedures involving animals were performed in compliance with the ethical standards of the Institutional Animal Care and Use Committee (IACUC) at the First Affiliated Hospital of Anhui Medical University. The mice were housed in a specific pathogen‐free environment at the same institution. The housing conditions included standard polypropylene cages with a 12‐h light/dark cycle, and were maintained at a stable temperature of 22 ± 2 °C and humidity of 55 ± 10%. Food and water were provided ad libitum. The mice were fed either a chow diet (Cat: D12450B, Research Diets), a high‐fat, high‐fructose (HFHfr) diet consisting of 40 kcal% fat (primarily palm oil), 20 kcal% fructose, and 2% cholesterol (Cat: D09100310, Research Diets), or a WD containing 40% of total calories from fat, 1.25% cholesterol, and 0.5% cholic acid (Cat: D12109C, Research Diets).

### Methods Details–Animal Experiments–Animal Experiment 1: HFHfr+*C. diff* FMT

The FMT process, which involved the utilization of the *C. diff* strain Bio‐18725, was carefully executed following established protocols. The *C. diff* strain was sourced from Beijing Biobw Company. For the cultivation of bacteria, an anaerobic environment was provided by the AnaeroPack system (Model MGC‐041, Mitsubishi Gas Chemical Company). The growth medium used was brain‐heart infusion (BHI) medium, procured from HiMedia Laboratories GmbH in Einhausen, Germany. This medium was further enriched with 5 g L^−1^ of yeast extract (sourced from Biolab Zrt., Budapest, Hungary) and 0.1% l‐cysteine (supplied by Acros Organics, Thermo Fisher Scientific, Waltham, MA). Bacterial cultures were incubated at optimal growth conditions for 24 h to reach an optical density of 1.5 in BHI medium. Subsequently, the cultures were aliquoted into 1 mL volumes to prepare for gavage administration into mice. Following a 12‐week regimen of a HFHfr diet (Research Diets, D12040701), the mice underwent biweekly oral gavage *C. diff* and continued on the high‐fat diet for a total of 16 weeks, as detailed in the protocol cited.^[^ ^3^ ^]^

### Animal Experiment 2: LCA+HFHfr Model

Following a 12‐week regimen of a HFHfr diet, mice were orally administered LCA a dose of 50 mg kg^−1^ dissolved in saline once a day along with HFHfr diet until 16 weeks.

### Animal Experiment 3: HFHfr Model

Mice (6–8 weeks old C57BL/6J, male) were randomly divided and treated with HFHfr diets (Research Diets, D12040701). Six mice from each group were euthanized after 0, 8, and 16 weeks. The liver specimen was collected for further histological evaluation and multi‐omics analysis.

### Animal Experiment 4: WD Model

This experiment followed a similar protocol to Animal experiment 3, but with the inclusion of a WD (Research Diets, D12079B).

### Animal Experiment 5: Therapeutic Model

To model MASH therapeutically, Rosa26‐LSL‐Cas9 knockin mice, aged 8 weeks, were treated with HFHfr diet for 8 weeks. Mice were processed with the initiation of either HFHfr+*C. diff* FMT or LCA+HFHfr model for four weeks. Then, the mice were randomized into three treatment groups: (1) *Bbox1* OE group (*n* = 8), injected with liver‐targeted Adeno‐Associated Virus 8 (AAV8) carrying the *Alb*‐*Bbox1* gene; (2) *Gpbar1* KO group (*n* = 8), receiving AAV8 with *Alb*‐Cre‐U6‐*Gpbar1* sgRNA; (3) *Trim21* KO group (*n* = 8), injected with AAV8 carrying the *Alb*‐Cre‐U6‐*Trim21* sgRNA. Each injection had a dose of 1 × 10^10^ viral genomes (vg) per mouse. Each mouse received 200 µL of the virus solution. Each receiving weekly intravenous tail vein injections, along with either HFHfr+*C. diff* FMT or LCA+HFHfr model, with continued proceeding until the 16th week.

### Animal Experiment 6: Prevention Model

In the prevention model for MASH, Rosa26‐LSL‐Cas9 knockin mice at 8 weeks of age were treated with HFHfr diet for 8 weeks. Then, the mice were randomized into three treatment groups, similar to the therapeutic model. Each group received tail vein injections weekly along with the treatment of the HFHfr+*C. diff* FMT or LCA+HFHfr model, with continued proceeding until the 16th week.

### Cell Culture and Reagents

The mouse liver cell line AML12, procured from the China Model Culture Center, Wuhan, and the Raw264.7 cell line and THP‐1 cell line, sourced from the Cell Bank of the Chinese Academy of Sciences, Shanghai, and HepRG cell line, sourced from Thermo Fisher Scientific were all authenticated as free from mycoplasma contamination. AML12, a mouse hepatocyte cell line, was cultured following standard protocols. The cells were maintained in Dulbecco's Modified Eagle Medium/Nutrient Mixture F‐12 Ham (DMEM/F12) supplemented with 10% fetal bovine serum (FBS) (Catalog No. 085‐150, WISENT), 1% insulin‐transferrin‐selenium (ITS), and 40 ng mL^−1^ dexamethasone. The culture medium was also supplemented with 100 U mL^−1^ penicillin and 100 µg mL^−1^ streptomycin to prevent bacterial contamination. Raw264.7 cells were cultured in DMEM, enriched with 1% penicillin‐streptomycin (Catalog No. BL505A, Biosharp Life Sciences) and 10% high‐quality FBS (Catalog No. 085‐150, WISENT). THP‐1 cells were cultured in RPMI 1640 medium (Catalog No. 350‐000‐CL, WISENT) supplemented with 10% fetal bovine serum, 1% penicillin‐streptomycin, and 0.05 × 10^−3^ m β‐mercaptoethanol (Catalog No. 444203, Sigma‐Aldrich). To induce differentiation into macrophages, THP‐1 cells were seeded at a density of 5 × 10^5^ cells mL^−1^ and treated with 100 ng mL^−1^ PMA (Catalog No. P8139, Sigma‐Aldrich) for 48 h. The medium was then replaced with fresh PMA‐free medium, and cells were allowed to rest for 24 h to complete differentiation. For polarization into M1 macrophages, differentiated macrophages were stimulated with 20 ng mL^−1^ IFN‐γ (Catalog No. UA040066, UA BIOSCIENCE) and 100 ng mL^−1^ LPS (Catalog No. L8880, Solarbio) for 24 h. HepRG cells were cultured according to the manufacturer's protocol (Catalog No. HPRGC10, Thermo Fisher Scientific). The cells were maintained in two different media sequentially: HepaRG Thaw, Plate & General Purpose Medium for initial thawing and plating, followed by HepaRG Maintenance/Metabolism Medium for long‐term maintenance and metabolic studies. All cell lines were maintained in a Thermo direct heat incubator at 37 °C and 5% CO_2_.

To study the effects of specific treatments, cells were exposed to INT777 (MCE, HY‐15677) at a concentration of 6 × 10^−6^ m, G/TLCA (GLCA, Sigma‐Aldrich, Catalog No. 474‐74‐8; TLCA, Sigma‐Aldrich, Catalog No. 516‐90‐5) at 50 × 10^−6^ m, and doxycycline hyclate (Sigma‐Aldrich, D5207) at 1 µg mL^−1^. These reagents were added to the culture medium for specified durations to investigate their impact on cell behavior and gene expression.

### 3D Cell Culture

For the 3D co‐culture of hepatocytes and macrophages (primary mouse hepatocyte, peritoneal macrophages [PMs] and HepRG, THP‐1), agarose microwells were used. Following manufacturer instructions, 330 µL of sterile saline containing 2% agarose (Catalog No. R0491, Thermo Fisher Scientific) was dispensed into sterile 3D Petri dishes (Catalog No. Z764051, Sigma‐Aldrich) to form the agarose microwells. After solidification, the microwells were transferred to a 24‐well plate. The microwells were equilibrated by adding 1 mL of culture medium to each well, followed by an incubation of over 15 min. Then, a mixture of 1.5 × 10^5^ liver cells and 1.5 × 10^5^ macrophages in 75 µL of culture medium was seeded into the agarose microwells. Seven days post‐seeding, cells were harvested for the preparation of O.C.T. (Catalog No. G6059‐110ML, Servicebio) or paraffin‐embedded, or preparing a single‐cell suspension, then removing macrophages by using flow cytometry with an F4/80 antibody.

### Biochemical Analysis

Serum cytokine concentrations in mice were quantified utilizing enzyme‐linked immunosorbent assay (ELISA) kits. These kits specifically targeted tumor necrosis factor (TNFα) (Cloud‐Clone, Catalog No. SEA133Mu) and monocyte chemoattractant protein‐1 (MCP1) (Cloud‐Clone, Catalog No. SEA087Mu). Serum levels of ALT were determined using an ADVIA 2400 Chemistry System analyzer (Siemens, Tarrytown, NY). All assays were conducted meticulously following the manufacturer's instructions. For the quantitative assessment of triglycerides (TG) and total cholesterol (TC) in liver samples, commercial assay kits (Wako, Catalog No. 290‐63701 for TG, and 294‐65801 for TC) were used, with strict adherence to the provided protocols.

### Histopathological Examination

Histological analyses were performed using multiple staining approaches. Hematoxylin and eosin (H&E) staining was applied to paraffin‐embedded sections to examine the general tissue architecture. Lipid droplets in frozen liver tissues, embedded in Tissue‐Tek O.C.T. Compound (Servicebio, G6059‐110ML), were visualized using Oil Red O staining. Masson's Trichrome staining (Solarbio, Catalog No. G1346) was used to evaluate liver fibrosis. All images were captured using an Olympus light microscope and were subjected to quantitative analysis utilizing Image‐Pro Plus 6.0 (Media Cybernetics) software.

### Multiple‐Colored Immunohistochemistry (mIHC)

Tissue samples, meticulously evaluated by two experienced pathologists, were sectioned into 5‐µm slices. Immunohistochemical staining was performed using specific antibodies against TGR5 (1:200, ab72608, Abcam), BBOX1 (1:200, 16099‐1‐AP, Proteintech), *CD36* (1:200, 18836‐1‐AP, Proteintech), *TRIM21* (1:200, 12108‐1‐AP, Proteintech), RIP3 (1:200, 17563‐1‐AP, Proteintech), CD68 (1:200, Catalog No. 26042, CST), F4/80 (1:200, Catalog No. 70076, CST), TNFα (1:200, Catalog No. 17590‐1‐AP, Proteintech), and OCTN2 (1:200, Catalog No. 16331‐1‐AP, Proteintech), followed by application of HRP‐conjugated secondary antibodies and various PPD dyes (Catalog No. 10234100050, PANOVUE). The identification of positive cells and subsequent analyses were automated using the Vectra 3 quantitative pathology imaging system and inForm software. inForm software was used, which uses an advanced machine‐learning‐based cell segmentation algorithm to classify and distinguish different cell types according to cell shape, size, and nuclear characteristics.

### Targeted Identification of LCA and Its Derivatives Concentration in MASLD Cohort

Targeted metabolomics analysis of bile acids had been reported in a previous work.^[^ ^2^ ^]^ A total of 550 Chinese adults with biopsy‐proven MASLD and 15 heathy volunteers as controls were included in the present study. All MASLD patients were consecutively recruited from 2016 to 2019 at the First Affiliated Hospital of Wenzhou Medical University in Wenzhou (China). This is a well‐characterized Prospective Epidemic Research Specifically of MASH (PERSONS) cohort. The inclusion and exclusion criteria and liver histology assessment were described elsewhere.^[^ ^1^ ^]^ Written informed consent was obtained from each subject before study participation. The research protocol was approved by the ethics committee of the First Affiliated Hospital of Wenzhou Medical University (2016‐246, December 01, 2016) and registered in the Chinese Clinical Trial Registry (ChiCTR‐EOC‐17013562).

### RNA‐Sequencing

Total RNA extraction, library construction, and sequencing for this study were outsourced to Gene *De novo* Biotechnology Co. (Guangzhou, China). In brief, RNA was extracted using Trizol reagent and underwent quality assessment. Eukaryotic mRNA was enriched using Oligo (dT) beads, followed by fragmentation using fragmentation buffer. Reverse transcription to cDNA was carried out using the NEBNext Ultra RNA Library Prep Kit for Illumina (Catalog No. 7530, New England Biolabs, Ipswich, MA). The resulting double‐stranded cDNA fragments were processed through end repair, A‐tailing, and ligation to Illumina sequencing adapters. After purification with AMPure XP Beads (1.0×) and size selection via agarose gel electrophoresis, PCR amplification was performed. The final cDNA library was sequenced on an Illumina NovaSeq 6000 platform.

### KEGG Pathway Enrichment Analysis

For KEGG pathway enrichment analysis, Fisher's exact test was utilized through a custom R script. KEGG pathway annotations for the selected genome's entire gene set were sourced from the KEGG database. Pathways were considered significantly enriched if they met a statistical significance threshold of a *P*‐value below 0.05.

### Gene Set Enrichment and Gene Set Variation Analysis

Gene set enrichment analysis (GSEA) and gene set variation analysis (GSVA) were conducted to assess pathway activity. Specifically, GSEA was executed using the Java GSEA platform version 3.0, utilizing gene sets defined for each known biological pathway sourced from the KEGG database. The “Signal2Noise” metric was used for ranking, and significance was determined based on nominal *P*‐values less than 0.05 and false discovery rate (FDR) values less than 0.25. Concurrently, GSVA was carried out using the GSVA R package version 1.32.0 to evaluate sample‐wise fluctuations in KEGG pathway activity.

### Quantitative Real‐Time PCR

Total RNA from cells and liver tissue was extracted using the Total RNA Isolation Kit (Catalog No. RC101‐01, Vazyme Biotech Co., Ltd., China), according to the manufacturer's guidelines. RNA concentration was quantified using a NanoDrop 2000C spectrophotometer (Thermo Fisher Scientific, Waltham, MA). For reverse transcription, 500 ng of purified RNA per sample was used, employing the Reverse Transcription Kit (Catalog No. R223, Vazyme Biotech Co., Ltd., China). Quantitative real‐time PCR was conducted on a QuantStudio 6 Flex Real‐Time PCR System (Applied Biosystems, CA, USA) using SYBR Green (Catalog No. Q221, Vazyme Co., Ltd., China). Primer sequences for qPCR, synthesized by GENEray Biotechnology (Shanghai, China), are listed in Table S1 (Supporting Information).

### Western Blot Analysis

Cellular and mouse liver tissue proteins were extracted using RIPA lysis buffer (Catalog No. P0013B, Beyotime) with added protease and phosphatase inhibitors (Catalog Nos. HY‐K0010, HY‐K0021, MCE). After centrifugation at 13000*g* for 20 min, protein concentration was determined using a BCA Protein Assay Kit (Catalog No. P0011, Beyotime). Protein samples underwent 10% SDS‐PAGE and were transferred to PVDF membranes (Catalog No. 03010040001, Roche). Membranes were blocked with 5% non‐fat milk (Catalog No. P0216‐1500g, Beyotime) and incubated overnight with primary antibodies. Following TBST washes, membranes were incubated with appropriate HRP‐conjugated secondary antibodies. Antibodys were performed using specific antibodies against *CD36* (1:1000, Catalog No. 18836‐1‐AP, Proteintech), *TRIM21* (1:1000, Catalog No. 12108‐1‐AP, Proteintech), TGR5 (1:1000, Catalog No. ab72608, Abcam), SHMT2 (1:1000, Catalog No. 11099‐1‐AP, Proteintech), BBOX1 (1:1000, Catalog No. 16099‐1‐AP, Proteintech), β‐actin (1:20000, Catalog No. 66009‐1‐Ig, Proteintech), and OCTN2 (1:1000, Catalog No. 16331‐1‐AP, Proteintech), followed by application of HRP‐conjugated secondary antibodies.

### Primary Mouse Hepatocyte Isolation and Cultivation

Primary hepatocytes were isolated from 8‐week‐old male *Gpbar1* ^fl/fl^×*Alb*‐Cre and *Gpbar1* ^fl/fl^ mice using a two‐step collagenase perfusion method, as described by Li et al.^[^ ^4^ ^]^ Mice were anesthetized with 3% pentobarbital sodium (90 mg kg^−1^, Catalog No. P3761, Sigma‐Aldrich), and the liver was perfused via the portal vein using specific media. Isolated hepatocytes were cultured under specified conditions and subjected to various treatments as outlined in the text.

### Isolation of Peritoneal Macrophages

PMs were induced with an intraperitoneal injection of 3% Brewer's yeast polysaccharide into mice, administered the day prior to harvest to boost cell number and activity, under sterile conditions. Mice were subsequently euthanized via CO_2_ asphyxiation and the abdomen sterilized with 75% ethanol. 5–10 mL of cold PBS or DMEM was injected into the peritoneum, the volume tailored to the size of the mouse. After gently massaging the abdomen to dislodge cells, the peritoneal fluid was collected into centrifuge tubes. The cells were pelleted by centrifugation at 300*g* for 5 min, followed by counting with a hemocytometer for further experimental use.

### Genotyping

*Lyz* Cre genotyping was performed by PCR using DNA from myeloid cells. The primers used were: F:AGTGCTGAAGTCCATAGATCGG and R: CTGATTCTCCTCATCACCAGG to detect the targeted (Cre) allele (543 bp). PCR was conducted with a 2×Taq Master Mix, starting with denaturation at 95 °C for 5 min, followed by 20 cycles of 98 °C for 30 s, 65 °C for 30 s (decreasing 0.5 °C cycle^−1^), and 72 °C for 45 s, with a final extension at 72 °C for 5 min*. Alb* Cre genotyping was performed by PCR using DNA from hepatocytes. The primers used were: F: ATTTGCCTGCATTACCGGTC and R: ATCAACGTTTTCTTTTCGG to detect the targeted (Cre) allele (350 bp). PCR was conducted with a 2×Taq Master Mix, starting with denaturation at 94 °C for 3 min, followed by 20 cycles of 94 °C for 20 s, 64 °C for 30 s (decreasing 0.5 °C cycle^−1^), and 72 °C for 35 s, with a final extension at 72 °C for 2 min. PCR products were analyzed by gel electrophoresis.

### Plasmid

The sgRNA sequences of mouse *Gpbar1* (F:TGGCTAGGGCTCTCACCTGG, R:CCAGGGTTGAGGGTACATCG) and *Trim21* (F:CAAAGGATCGGAGACAAGTG, R:GATGATGAGCCATGAGTTGG) sequences were cloned into the lentiCRISPR v2‐purinomycin vector using BfuAI and BsmBI to construct CRISPR plasmids. For overexpression of target genes, mouse *Bbox1* genes were cloned into the pCDH‐CMV‐MCS‐EF1‐Puro vector. *Bbox1* variants with specific mutations (D282E or K283N) were synthesized and similarly cloned by Corues Biotechnology (Nanjing, China). Plasmids were transfected using ExFect Transfection Reagent (Vazyme, T101‐01) and verified by Western blotting.

AAV constructs (AAV8‐*Alb*‐*Bbox1*, AAV8‐*Alb*‐Cre‐U6‐*Gpbar1* sgRNAs, and AAV8‐*Alb*‐Cre‐U6‐*Trim21* sgRNAs) were created using the pAAV‐TBG‐CBh‐ 3xFLAG vector. CDS of mouse *Bbox1*, sgRNA of *Trim21*, and sgRNA of *Gpbar1* were inserted and cloned in DH5α. Post‐sequence verification, 293 cells were transfected with these plasmids, pAAV‐RC, and pHelper plasmid. AAV particles were purified (Catalog No. V1469, Biomiga) and quantified using PCR. For in vivo administration, the AAV8 vectors were delivered via tail vein injections at a dose of 1 × 10^10^ vg per mouse. Each mouse received 200 µL of the virus solution. Injections were performed once per week for four consecutive weeks to ensure sustained expression of the overexpressed genes or knockout constructs in hepatocytes. Virus titers were verified using a quantitative PCR‐based assay.

For the Tet‐off system in AML12 cells, *Bbox1* was cloned into the pTRE‐Tight Tet‐off vector. Stable cell lines were established using G418 antibiotic selection, ensuring conditional BBOX1 expression controlled by Doxycycline in Tet‐off conditions. This system's efficacy was validated via qRT‐PCR and WB.

### Protein‐Docking

*CD36* and TGR5 structures were predicted using Alphafold2, while BBOX1 and *TRIM21* structures were obtained from RCSB PDB (PDB ID: 4BG1, 7BBD). Post‐preprocessing, initial conformations were adjusted through rigid docking on ClusPro 2.0, followed by flexible docking using RosettaDock. Interprotein interactions were analyzed using Ligplot, and final protein conformations were visualized with Pymol software.

### Co‐Immunoprecipitation and LC/Mass Spectrometry

For immunoprecipitation assays, cells were lysed using Co‐IP lysis buffer (Catalog No. P0013, Beyotime) and centrifuged at 12000×*g* for 20 min at 4 °C to pellet the lysate. Protein concentration was standardized to 1 mg mL^−1^ based on BCA assay results. Protein AG beads (Beyotime, P2108) were pre‐equilibrated with 1 mL of wash buffer (Beyotime, ST661) and incubated overnight at 4 °C with 1 mg of lysate along with specific antibodies (5 µg of BBOX1(16099‐1‐AP, Proteintech), *CD36*(18836‐1‐AP, Proteintech), or *TRIM21*(12108‐1‐AP, Proteintech)) or a rabbit IgG isotype control. The samples were then subjected to electrophoresis, followed by staining with Coomassie Blue Fast Staining Solution (Beyotime, P0017). Mass spectrometry analysis was performed (Shanghai Applied Protein Technology) and the immunoprecipitated proteins were further assessed through immunoblotting using appropriate antibodies.

### Statistical Analysis

In this study, data are presented as the mean ± standard Deviation (SD). The generation of all bar plots was accomplished using GraphPad Prism 9.0 (GraphPad Software, La Jolla, CA). To determine the distribution of the samples, normality tests were performed. For the statistical comparison of normally distributed variables, one‐way ANOVA was used to analyze more than two groups. Two‐way ANOVA was used to analyze data involving two independent factors. These analyses were conducted using SPSS 26.0 (IBM SPSS, Chicago, IL). In addition, Spearman's rank correlation coefficient was used to assess the relationship between variables, with graphical visualizations of these correlations produced in R studio (RStudio, Boston, MA).

## Conflict of Interest

The authors declare no conflict of interest.

## Author Contributions

S.L., M.L., L.J., and B.Z. contributed equally to this work. R.J., G.X., Y.N., Y.Y., and S.L. designed the research. S.L., M.L., J.L., B.Z., Y.F., X.W., and Z.H. performed the experiments and analyzed the data. M.L., J.L., X.W., and Y.F. participated in mouse feeding and experiments. S.L., M.L., and B.Z. were responsible for data statistics. S.L. and Y.F. validated the manuscript and data. R.J., G.X., Y.N., Y.Y., and S.L. drafted and revised the manuscript. R.J., G.X., Y.N., Y.Y., and S.L. designed, supervised, wrote, and revised the paper.

## Supporting information



Supporting Information

## Data Availability

The data that support the findings of this study are available from the corresponding author upon reasonable request.
