# Supplementary material for: Conjugated Lithocholic Acid Activates Hepatic TGR5 to Promote Lipotoxicity and MASLD‐MASH Transition by Disrupting Carnitine Biosynthesis
Source: Adv Sci (Weinh). 2025 May 8;12(20):2410602. doi: 10.1002/advs.202410602 (PMC12120702; doi:10.1002/advs.202410602)
Supplement: Supplementary file 1 — Supporting Information [file ADVS-12-2410602-s001.docx]

**Supplementary Data**

**Materials and methods**

**Human study**

Participants for this study were enrolled between January 2021 and June 2022 at the liver clinic associated with the First Affiliated Hospital of Anhui Medical University, Gulou Hospital, and People’s Hospital of Taizhou. Inclusion criteria were adults between the ages of 35 and 65 years, and all participants were categorized as Healthy control, MASLD, and MASH, as evidenced by histopathological analysis of liver biopsy specimens. Liver histopathology for all study groups was independently verified by a pair of medical society-certified pathologists to ensure the validity of inclusion. Individuals with one of the following conditions were excluded from participation in this study: documented history of chronic viral hepatitis, excessive alcohol consumption, or other diagnoses of liver disease. Liver biopsy samples were collected using an ultrasound-guided technique. A venous blood sample was also collected from each participant for further analysis. All collected samples were rapidly frozen and preserved in liquid nitrogen and then stored at -80 degrees Celsius until analysis. The study was approved by the Institutional Ethics Committee of the First Affiliated Hospital of Anhui Medical University to ensure compliance with ethical standards and participant safety.

Cohort from Wenzhou is a well-characterized Prospective Epidemic Research Specifically of MASH (PERSONS) cohort. All MASLD patients in this study were consecutively recruited from 2016 to 2019 at the First Affiliated Hospital of Wenzhou Medical University in Wenzhou (China). The inclusion and exclusion criteria and liver histology assessment have been described extensively elsewhere^[1]^. Written informed consent was obtained from each subject before study participation. The research protocol was approved by the ethics committee of the First Affiliated Hospital of Wenzhou Medical University (2016-246, 1 December 2016) and registered in the Chinese Clinical Trial Registry (ChiCTR-EOC-17013562). A total of 550 Chinese adults with biopsy-proven MASLD and 15 heathy volunteers as controls were included in the present study. Serum samples were collected for targeted metabolomics analysis of bile acids that had been reported in our previous work^[2]^.

**Mouse Models**

C57BL/6 mice, *Gpbar1*^fl/fl^ mice, *Alb-*Cre mice, and *Lyz*-Cre mice were purchased from GemPharmatech, while Rosa26-LSL-Cas9 knockin mice were obtained from The Jackson Laboratory. To generate hepatocyte-specific deletions of *Gpbar1*, *Gpbar*1^fl/fl^ mice were crossed with *Alb*-Cre mice. To generate myeloid-specific deletions of *Gpbar1*, *Gpbar*1^fl/fl^ mice were crossed with *Lyz*-Cre mice. To generate myeloid-specific *Gpbar1* knockout mice with Cas9, we crossed myeloid-specific *Gpbar1* knockout mice (*Gpbar1*^fl/fl^ × *Lyz*-Cre) with Rosa26-LSL-Cas9 knockin mice. All procedures involving animals were performed in compliance with the ethical standards of the Institutional Animal Care and Use Committee (IACUC) at the First Affiliated Hospital of Anhui Medical University. The mice were housed in a specific pathogen-free environment at the same institution. The housing conditions included standard polypropylene cages with a 12-hour light/dark cycle, and were maintained at a stable temperature of 22 ± 2℃ and humidity of 55 ± 10%. Food and water were provided ad libitum. The mice were fed either a chow diet (Cat: D12450B, Research Diets), a high-fat, high-fructose (HFHfr) diet consisting of 40 kcal% fat (primarily palm oil), 20 kcal% fructose, and 2% cholesterol (Cat: D09100310, Research Diets), or a Western diet (WD) containing 40% of total calories from fat, 1.25% cholesterol, and 0.5% cholic acid (Cat: D12109C, Research Diets).

**Methods details**

**Animal experiments**

**Animal experiment 1: HFHfr+*C.diff*** **FMT**

The fecal microbiota transplantation (FMT) process, which involved the utilization of the *Clostridium difficile* (*C.diff*) strain Bio-18725, was carefully executed following established protocols. The *C. diff* strain was sourced from Beijing Biobw Company. For the cultivation of bacteria, an anaerobic environment was provided by the AnaeroPack system (Model MGC-041, Mitsubishi Gas Chemical Company). The growth medium employed was Brain-Heart Infusion (BHI) Medium, procured from HiMedia Laboratories GmbH in Einhausen, Germany. This medium was further enriched with 5 g/L of yeast extract (sourced from Biolab Zrt., Budapest, Hungary) and 0.1% L-cysteine (supplied by Acros Organics, Thermo Fisher Scientific, Waltham, MA, USA). Bacterial cultures were incubated at optimal growth conditions for 24 hours to reach an optical density of 1.5 in BHI medium. Subsequently, the cultures were aliquoted into 1 ml volumes to prepare for gavage administration into mice. Following a 12-week regimen of a HFHfr diet (Research Diets, D12040701), the mice underwent biweekly oral gavage *C. diff* and continued on the high-fat diet for a total of 16 weeks, as detailed in the protocol cited^[3]^.

**Animal experiment 2: LCA+HFHfr model**

Following a 12-week regimen of a HFHfr diet, mice were orally administered LCA a dose of 50 mg/kg dissolved in saline once a day along with HFHfr diet until 16w.

**Animal experiment 3: HFHfr model**

Mice (6-8 weeks old C57BL/6J, male) were randomly divided and treated with HFHfr diets (Research Diets, D12040701). Six mice from each group were euthanized after 0-week, 8-week, and 16-week. The liver specimen was collected for further histological evaluation and multi-omics analysis.

**Animal experiment 4: WD model**

This experiment followed a similar protocol to Animal experiment 3, but with the inclusion of a Western Diet (WD)(Research Diets,D12079B).

**Animal experiment 5: Therapeutic Model**

To model MASH therapeutically, Rosa26-LSL-Cas9 knockin mice, aged 8 weeks, were treated with HFHfr diet for 8 weeks. Mice were processed with the initiation of either HFHfr+*C.diff* FMT or LCA+HFHfr model for four weeks. Then, the mice were randomized into three treatment groups: (1) *Bbox1* OE group (n=8), injected with liver-targeted Adeno-Associated Virus 8 (AAV8) carrying the *Alb*-*Bbox1* gene; (2) *Gpbar1* KO group (n=8), receiving AAV8 with *Alb*-Cre-U6-*Gpbar1* sgRNA; (3) *Trim21* KO group (n=8), injected with AAV8 carrying the *Alb*-Cre-U6-*Trim21* sgRNA. Each injection had a dose of 1 × 10^10^ viral genomes (vg) per mouse. Each mouse received 200 µL of the virus solution.. Each receiving weekly intravenous tail vein injections, along with either HFHfr+*C.diff* FMT or LCA+HFHfr model, with continued proceeding until the 16th week.

**Animal experiment 6: Prevention Model**

In the prevention model for MASH, Rosa26-LSL-Cas9 knockin mice at 8 weeks of age were treated with HFHfr diet for 8 weeks.Then, the mice were randomized into three treatment groups, similar to the therapeutic model. Each group received tail vein injections weekly along with the treatment of the HFHfr+*C.diff* FMT or LCA+HFHfr model, with continued proceeding until the 16th week.

**Cell Culture and reagents**

The mouse liver cell line AML12, procured from the China Model Culture Center, Wuhan, and the Raw264.7 cell line and THP-1 cell line, sourced from the Cell Bank of the Chinese Academy of Sciences, Shanghai, and HepRG cell line, sourced from Thermo Fisher Scientific were all authenticated as free from mycoplasma contamination. AML12, a mouse hepatocyte cell line, was cultured following standard protocols. The cells were maintained in Dulbecco's Modified Eagle Medium/Nutrient Mixture F-12 Ham (DMEM/F12) supplemented with 10% fetal bovine serum (FBS) (Catalog No: 085-150, WISENT), 1% insulin-transferrin-selenium (ITS), and 40 ng/ml dexamethasone. The culture medium was also supplemented with 100 U/ml penicillin and 100 µg/ml streptomycin to prevent bacterial contamination. Raw264.7 cells were cultured in DMEM, enriched with 1% penicillin-streptomycin (Catalog No: BL505A, Biosharp Life Sciences) and 10% high-quality FBS (Catalog No: 085-150, WISENT). THP-1 cells were cultured in RPMI 1640 medium(Catalog No: 350-000-CL, WISENT) supplemented with 10% fetal bovine serum, 1% penicillin-streptomycin, and 0.05 mM β-mercaptoethanol (Catalog No: 444203, Sigma-Aldrich). To induce differentiation into macrophages, THP-1 cells were seeded at a density of 5×10^5^ cells/mL and treated with 100 ng/mL PMA(Catalog No: P8139, Sigma-Aldrich) for 48 hours. The medium was then replaced with fresh PMA-free medium, and cells were allowed to rest for 24 hours to complete differentiation. For polarization into M1 macrophages, differentiated macrophages were stimulated with 20 ng/mL IFN-γ (Catalog No: UA040066, UA BIOSCIENCE) and 100 ng/mL LPS (Catalog No: L8880, Solarbio) for 24 hours. HepRG cells were cultured according to the manufacturer's protocol (Catalog No: HPRGC10, Thermo Fisher Scientific). The cells were maintained in two different media sequentially: HepaRG™ Thaw, Plate & General Purpose Medium for initial thawing and plating, followed by HepaRG™ Maintenance/Metabolism Medium for long-term maintenance and metabolic studies. All cell lines were maintained in a Thermo direct heat incubator at 37°C and 5% CO2.

To study the effects of specific treatments, cells were exposed to INT777 (MCE, HY-15677) at a concentration of 6 µM, G/TLCA (GLCA, Sigma-Aldrich, Catalog No: 474-74-8; TLCA, Sigma-Aldrich, Catalog No:516-90-5) at 50 µM, and Doxycycline hyclate (Sigma-Aldrich, D5207) at 1 µg/ml. These reagents were added to the culture medium for specified durations to investigate their impact on cell behavior and gene expression.

**3D Cell Culture**

For the 3D co-culture of hepatocytes and macrophages (Primary Mouse Hepatocyte, Peritoneal macrophages and HepRG, THP-1), agarose microwells were employed. Following manufacturer instructions, 330 μl of sterile saline containing 2% agarose (Catalog No: R0491, Thermo Fisher Scientific) was dispensed into sterile 3D Petri dishes (Catalog No: Z764051, Sigma-Aldrich) to form the agarose microwells. After solidification, the microwells were transferred to a 24-well plate. The microwells were equilibrated by adding 1 ml of culture medium to each well, followed by an incubation of over 15 minutes. Then, a mixture of 1.5×10^5^ liver cells and 1.5×10^5^ macrophages in 75 μl of culture medium was seeded into the agarose microwells. Seven days post-seeding, cells were harvested for the preparation of O.C.T. (Catalog No: G6059-110ML, Servicebio) or paraffin-embedded, or preparing a single-cell suspension, then removing macrophages by using flow cytometry with an F4/80 antibody.

**Biochemical Analysis**

Serum cytokine concentrations in mice were quantified utilizing Enzyme-Linked Immunosorbent Assay (ELISA) kits. These kits specifically targeted Tumor Necrosis Factor (TNFα) (Cloud-Clone, Catalog No. SEA133Mu) and Monocyte Chemoattractant Protein-1 (MCP1) (Cloud-Clone, Catalog No. SEA087Mu). Serum levels of Alanine Aminotransferase (ALT) were determined using an ADVIA 2400 Chemistry System analyzer (Siemens, Tarrytown, NY, USA). All assays were conducted meticulously following the manufacturer's instructions. For the quantitative assessment of triglycerides (TG) and total cholesterol (TC) in liver samples, commercial assay kits (Wako, Catalog No: 290-63701 for TG, and 294-65801 for TC) were employed, with strict adherence to the provided protocols.

**Histopathological Examination**

Histological analyses were performed employing multiple staining approaches. Hematoxylin and Eosin (H&E) staining was applied to paraffin-embedded sections to examine the general tissue architecture. Lipid droplets in frozen liver tissues, embedded in Tissue-Tek O.C.T. Compound (Servicebio, G6059-110ML), were visualized using Oil Red O staining. Masson's Trichrome staining (Solarbio, Catalog No. G1346) was used to evaluate liver fibrosis. All images were captured using an Olympus light microscope and were subjected to quantitative analysis utilizing Image-Pro Plus 6.0 (Media Cybernetics) software.

**Multiple-Colored Immunohistochemistry (mIHC)**

Tissue samples, meticulously evaluated by two experienced pathologists, were sectioned into 5-μm slices. Immunohistochemical staining was performed using specific antibodies against TGR5 (1:200, ab72608, Abcam), BBOX1 (1:200, 16099-1-AP, Proteintech), CD36 (1:200, 18836-1-AP, Proteintech), TRIM21 (1:200, 12108-1-AP, Proteintech), RIP3 (1:200, 17563-1-AP, Proteintech), CD68(1:200, Catalog No: 26042, CST), F4/80(1:200, Catalog No: 70076, CST), TNFα(1:200, Catalog No: 17590-1-AP, Proteintech) and OCTN2 (1:200, Catalog No: 16331-1-AP, Proteintech), followed by application of HRP-conjugated secondary antibodies and various PPD dyes (Catalog No: 10234100050, PANOVUE). The identification of positive cells and subsequent analyses were automated using the Vectra 3 quantitative pathology imaging system and inForm software. We utilized inForm software, which employs an advanced machine-learning-based cell segmentation algorithm to classify and distinguish different cell types according to cell shape, size, and nuclear characteristics.

**Targeted identification of LCA and its derivatives concentration in MASLD cohort**

Targeted metabolomics analysis of bile acids had been reported in our previous work^[2]^. A total of 550 Chinese adults with biopsy-proven MASLD and 15 heathy volunteers as controls were included in the present study. All MASLD patients were consecutively recruited from 2016 to 2019 at the First Affiliated Hospital of Wenzhou Medical University in Wenzhou (China). This is a well-characterized Prospective Epidemic Research Specifically of MASH (PERSONS) cohort. The inclusion and exclusion criteria, and liver histology assessment have been described elsewhere^[1]^. Written informed consent was obtained from each subject before study participation. The research protocol was approved by the ethics committee of the First Affiliated Hospital of Wenzhou Medical University (2016-246, 1 December 2016) and registered in the Chinese Clinical Trial Registry (ChiCTR-EOC-17013562).

**RNA-Sequencing**

Total RNA extraction, library construction, and sequencing for this study were outsourced to Gene *De novo* Biotechnology Co. (Guangzhou, China). In brief, RNA was extracted using Trizol reagent and underwent quality assessment. Eukaryotic mRNA was enriched using Oligo (dT) beads, followed by fragmentation using fragmentation buffer. Reverse transcription to cDNA was carried out using the NEBNext Ultra RNA Library Prep Kit for Illumina (Catalog No. 7530, New England Biolabs, Ipswich, MA, USA). The resulting double-stranded cDNA fragments were processed through end repair, A-tailing, and ligation to Illumina sequencing adapters. After purification with AMPure XP Beads (1.0X) and size selection via agarose gel electrophoresis, PCR amplification was performed. The final cDNA library was sequenced on an Illumina NovaSeq 6000 platform.

**KEGG Pathway Enrichment Analysis**

For KEGG pathway enrichment analysis, Fisher’s exact test was utilized through a custom R script. KEGG pathway annotations for the selected genome’s entire gene set were sourced from the KEGG database. Pathways were considered significantly enriched if they met a statistical significance threshold of a P-value below 0.05.

**Gene Set Enrichment and Gene Set Variation Analysis**

Gene set enrichment analysis (GSEA) and gene set variation analysis (GSVA) were conducted to assess pathway activity. Specifically, GSEA was executed using the Java GSEA platform version 3.0, utilizing gene sets defined for each known biological pathway sourced from the KEGG database. The 'Signal2Noise' metric was used for ranking, and significance was determined based on nominal P values less than 0.05 and false discovery rate (FDR) values less than 0.25. Concurrently, GSVA was carried out using the GSVA R package version 1.32.0 to evaluate sample-wise fluctuations in KEGG pathway activity.

**Quantitative Real-Time PCR**

Total RNA from cells and liver tissue was extracted using the Total RNA Isolation Kit (Catalog No: RC101-01, Vazyme Biotech Co., Ltd., China), according to the manufacturer's guidelines. RNA concentration was quantified using a NanoDrop 2000C spectrophotometer (Thermo Fisher Scientific, Waltham, MA, USA). For reverse transcription, 500ng of purified RNA per sample was used, employing the Reverse Transcription Kit (Catalog No: R223, Vazyme Biotech Co., Ltd., China). Quantitative real-time PCR was conducted on a QuantStudio 6 Flex Real-Time PCR System (Applied Biosystems, CA, USA) using SYBR Green (Catalog No: Q221, Vazyme Co., Ltd., China). Primer sequences for qPCR, synthesized by GENEray Biotechnology (Shanghai, China), are listed in Table S1.

**Western Blot Analysis**

Cellular and mouse liver tissue proteins were extracted using RIPA lysis buffer (Catalog No: P0013B, Beyotime) with added protease and phosphatase inhibitors (Catalog Nos: HY-K0010, HY-K0021, MCE). After centrifugation at 13,000g for 20 minutes, protein concentration was determined using a BCA Protein Assay Kit (Catalog No: P0011, Beyotime). Protein samples underwent 10% SDS-PAGE and were transferred to PVDF membranes (Catalog No: 03010040001, Roche). Membranes were blocked with 5% non-fat milk (Catalog No: P0216-1500g, Beyotime) and incubated overnight with primary antibodies. Following TBST washes, membranes were incubated with appropriate HRP-conjugated secondary antibodies. Antibodys were performed using specific antibodies against CD36 (1:1000, Catalog No:18836-1-AP, Proteintech), TRIM21 (1:1000, Catalog No:12108-1-AP, Proteintech), TGR5 (1:1000, Catalog No:ab72608, Abcam), SHMT2 (1:1000, Catalog No: 11099-1-AP, Proteintech), BBOX1 (1:1000, Catalog No: 16099-1-AP, Proteintech), β-actin (1:20000, Catalog No: 66009-1-Ig, Proteintech) and OCTN2 (1:1000, Catalog No: 16331-1-AP, Proteintech), followed by application of HRP-conjugated secondary antibodies.

**Primary Mouse Hepatocyte Isolation and Cultivation**

Primary hepatocytes were isolated from 8-week-old male *Gpbar1*^fl/fl^×*Alb*-Cre and *Gpbar1*^fl/fl^ mice using a two-step collagenase perfusion method, as described in Li et al., 2010^[4]^. Mice were anesthetized with 3% pentobarbital sodium (90 mg/kg, Catalog No: P3761, Sigma-Aldrich), and the liver was perfused via the portal vein using specific media. Isolated hepatocytes were cultured under specified conditions and subjected to various treatments as outlined in the text.

**Isolation of Peritoneal Macrophages**

Peritoneal macrophages (PMs) were induced with an intraperitoneal injection of 3% Brewer's yeast polysaccharide into mice, administered the day prior to harvest to boost cell number and activity, under sterile conditions. Mice were subsequently euthanized via CO2 asphyxiation and the abdomen sterilized with 75% ethanol. We injected 5-10 mL of cold PBS or DMEM into the peritoneum, the volume tailored to the size of the mouse. After gently massaging the abdomen to dislodge cells, the peritoneal fluid was collected into centrifuge tubes. The cells were pelleted by centrifugation at 300g for 5 minutes, followed by counting with a hemocytometer for further experimental use.

**Genotyping**

*Lyz* Cre genotyping was performed by PCR using DNA from myeloid cells. The primers used were: F:AGTGCTGAAGTCCATAGATCGG and R: CTGATTCTCCTCATCACCAGG to detect the targeted (Cre) allele (543 bp). PCR was conducted with a 2×Taq Master Mix, starting with denaturation at 95°C for 5 minutes, followed by 20 cycles of 98°C for 30 seconds, 65°C for 30 seconds (decreasing 0.5°C per cycle), and 72°C for 45 seconds, with a final extension at 72°C for 5 minutes*. Alb* Cre genotyping was performed by PCR using DNA from hepatocytes. The primers used were: F: ATTTGCCTGCATTACCGGTC and R: ATCAACGTTTTCTTTTCGG to detect the targeted (Cre) allele (350 bp). PCR was conducted with a 2×Taq Master Mix, starting with denaturation at 94°C for 3 minutes, followed by 20 cycles of 94°C for 20 seconds, 64°C for 30 seconds (decreasing 0.5°C per cycle), and 72°C for 35 seconds, with a final extension at 72°C for 2 minutes. PCR products were analyzed by gel electrophoresis.

**Plasmid**

The sgRNA sequences of mouse *Gpbar1* (F:TGGCTAGGGCTCTCACCTGG, R:CCAGGGTTGAGGGTACATCG) and *Trim21* (F:CAAAGGATCGGAGACAAGTG, R:GATGATGAGCCATGAGTTGG) sequences were cloned into the lentiCRISPR v2-purinomycin vector using BfuAI and BsmBI to construct CRISPR plasmids. For overexpression of target genes, mouse *Bbox1* genes were cloned into the pCDH-CMV-MCS-EF1-Puro vector. *Bbox1* variants with specific mutations (D282E or K283N) were synthesized and similarly cloned by Corues Biotechnology (Nanjing, China). Plasmids were transfected using ExFect Transfection Reagent (Vazyme, T101-01) and verified by Western blotting.

AAV constructs (AAV8-*Alb*-*Bbox1*, AAV8-*Alb*-Cre-U6-*Gpbar1* sgRNAs, and AAV8-*Alb*-Cre-U6-*Trim21* sgRNAs) were created using the pAAV-TBG-CBh- 3xFLAG vector. CDS of mouse *Bbox1*, sgRNA of *Trim21*, and sgRNA of *Gpbar1* were inserted and cloned in DH5α. Post-sequence verification, 293 cells were transfected with these plasmids, pAAV-RC, and pHelper plasmid. AAV particles were purified (Catalog No:V1469, Biomiga) and quantified using PCR. For *in vivo* administration, the AAV8 vectors were delivered via tail vein injections at a dose of 1 × 10^10^ viral genomes (vg) per mouse. Each mouse received 200 µL of the virus solution. Injections were performed once per week for 4 consecutive weeks to ensure sustained expression of the overexpressed genes or knockout constructs in hepatocytes. Virus titers were verified using a quantitative PCR-based assay.

For the Tet-off system in AML12 cells, *Bbox1* was cloned into the pTRE-Tight Tet-off vector. Stable cell lines were established using G418 antibiotic selection, ensuring conditional BBOX1 expression controlled by Doxycycline in Tet-off conditions. This system's efficacy was validated via qRT-PCR and WB.

**Protein-Docking**

CD36 and TGR5 structures were predicted using Alphafold2, while BBOX1 and TRIM21 structures were obtained from RCSB PDB (PDB ID: 4BG1, 7BBD). Post-preprocessing, initial conformations were adjusted through rigid docking on ClusPro 2.0, followed by flexible docking using RosettaDock. Interprotein interactions were analyzed using Ligplot, and final protein conformations were visualized with Pymol software.

**Co-Immunoprecipitation and LC/Mass Spectrometry**

For immunoprecipitation assays, cells were lysed using Co-IP lysis buffer (Catalog No: P0013, Beyotime) and centrifuged at 12,000×g for 20 minutes at 4°C to pellet the lysate. Protein concentration was standardized to 1 mg/mL based on BCA assay results. Protein AG beads (Beyotime, P2108) were pre-equilibrated with 1 mL of wash buffer (Beyotime, ST661) and incubated overnight at 4°C with 1 mg of lysate along with specific antibodies (5 μg of BBOX1(16099-1-AP, Proteintech), CD36(18836-1-AP, Proteintech), or TRIM21(12108-1-AP, Proteintech)) or a rabbit IgG isotype control. The samples were then subjected to electrophoresis, followed by staining with Coomassie Blue Fast Staining Solution (Beyotime, P0017). Mass spectrometry analysis was performed (Shanghai Applied Protein Technology) and the immunoprecipitated proteins were further assessed through immunoblotting using appropriate antibodies.

**Statistical Analysis**

In this study, data are presented as the mean ± standard Deviation (SD). The generation of all bar plots was accomplished using GraphPad Prism 9.0 (GraphPad Software, La Jolla, CA, USA). To determine the distribution of the samples, normality tests were performed. For the statistical comparison of normally distributed variables, One-way ANOVA was used to analyze more than two groups. Two-way ANOVA was used to analyze data involving two independent factors. These analyses were conducted using SPSS 26.0 (IBM SPSS, Chicago, IL, USA). Additionally, Spearman's rank correlation coefficient was used to assess the relationship between variables., with graphical visualizations of these correlations produced in R studio (RStudio, Boston, MA, USA).

**Supplementary Figures**

**
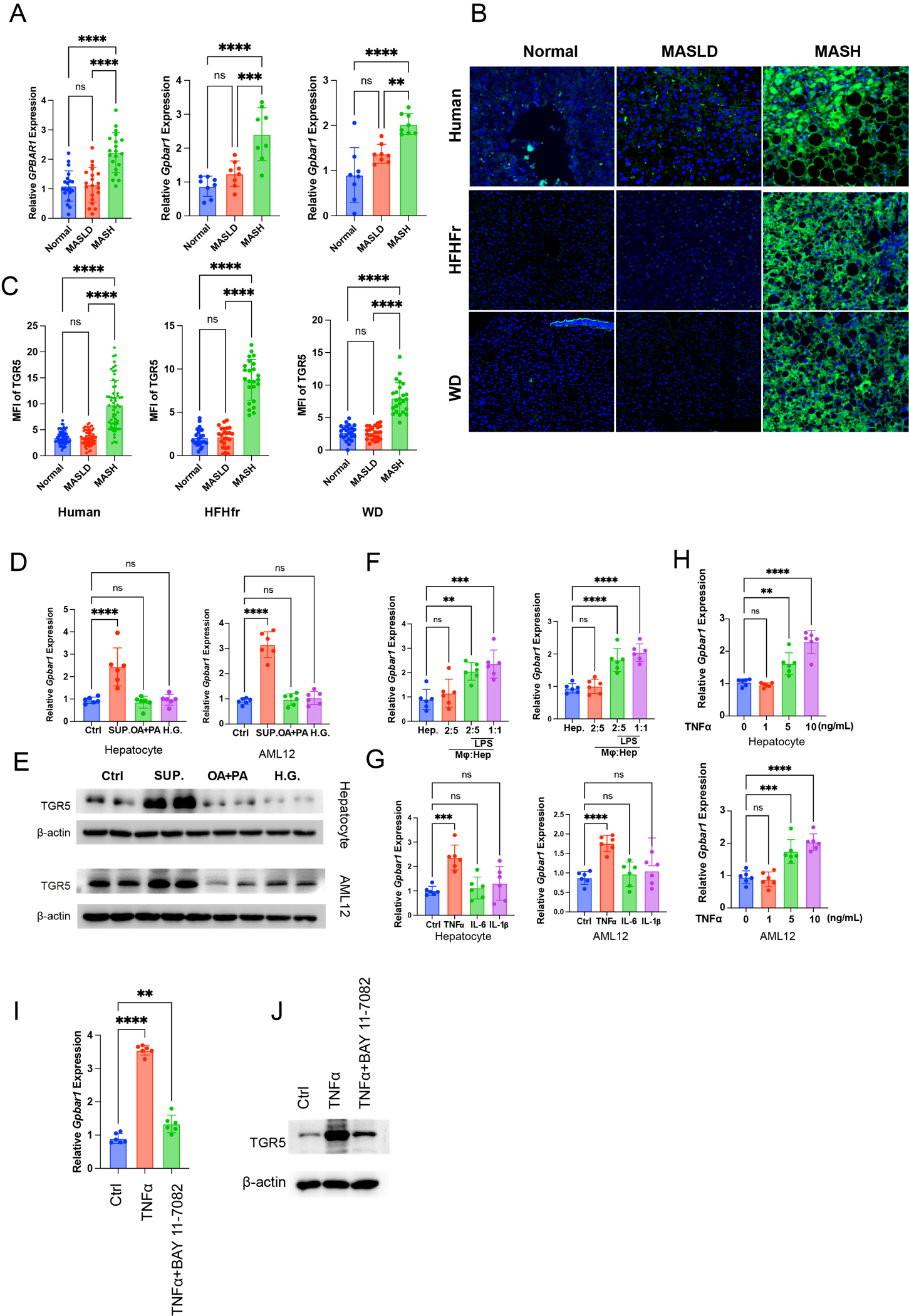

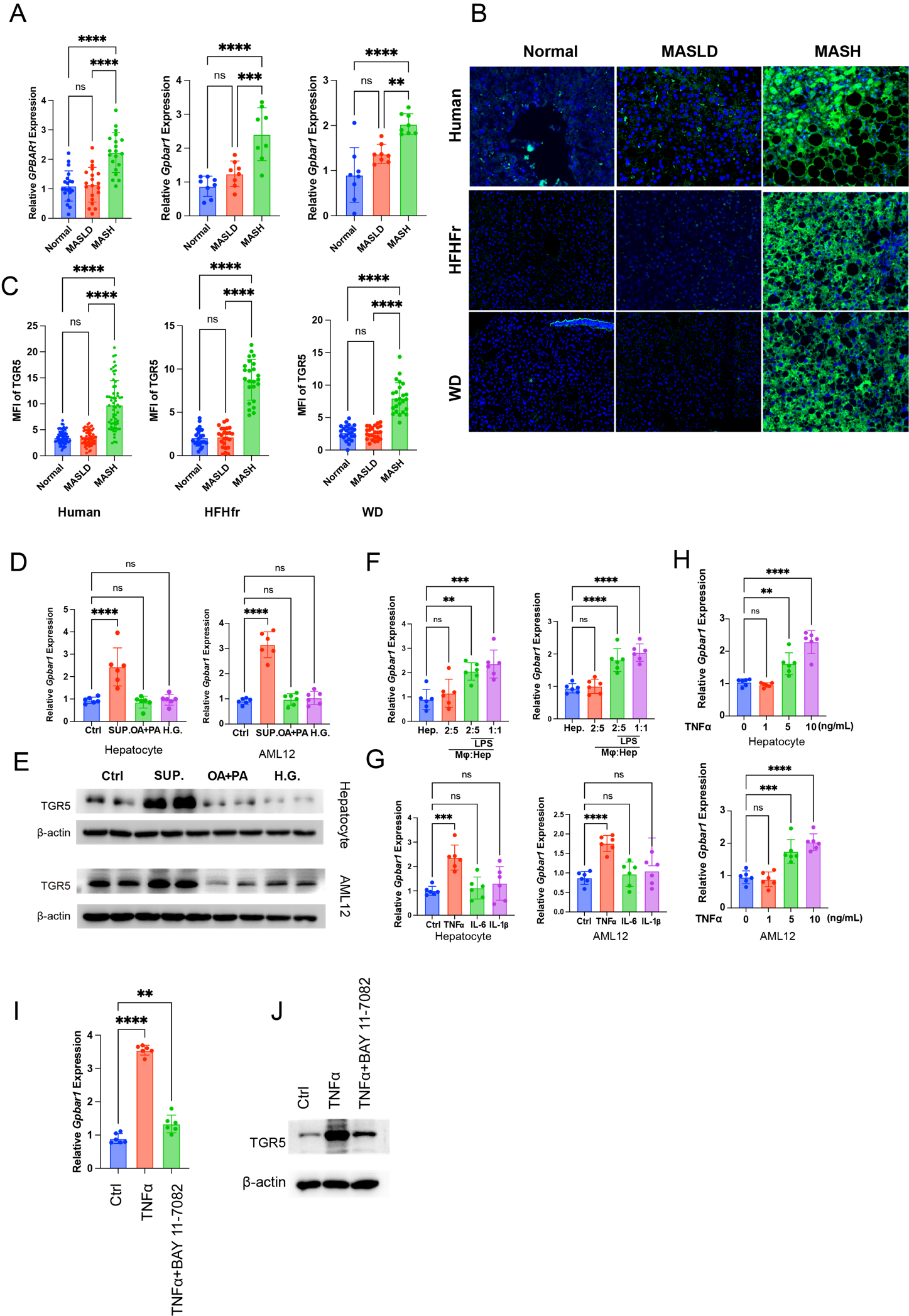
**

**Figure S1**

(A) Relative mRNA expression of *GPBAR1* in human liver samples and mouse liver samples in two different models at different MASLD stages (Normal n=60, MASLD n=60, MASH n=60).

(B) and (C) mIHC staining and mean fluorescence intensity (MFI) qualification of TGR5 expression in liver sections from human samples and mouse liver samples in two different models (Normal n=24, MASLD n=24, MASH n=24).

(D) Relative mRNA expression of *Gpbar1* in hepatocytes and AML12 with the treatment of supernatant of LPS-stimulated macrophages (SUP.), high-fat (OA+PA), and high glucose (H.G.) conditions, each group n=6.

(E) Western-blot (WB) dynamically monitors the protein expression of TGR5 in hepatocytes and AML12 after the treatment shown in the figure, with β-actin as the internal control.

(F) Relative mRNA expression of *Gpbar1* in hepatocytes and AML12 after co-culturing with primary macrophages and Raw264.7 at different ratios, with or without LPS stimulation, each group n=6.

(G) and (H) Relative mRNA expression of *Gpbar1* in hepatocytes and AML12 with different treatments and with different doses of TNFα, each group n=6.

(I) and (J) Expression of TGR5 at the transcriptional and protein levels in response to NF-κB inhibitors, each group n=6. (A-I) Statistical significance was determined using one-way ANOVA test with Dunnett adjustment.

Data are presented as mean ± SD. All experiments were performed in triplicate. ***p*<0.01, ****p*<0.0002, *****p*<0.0001, ns: *p*>0.05.


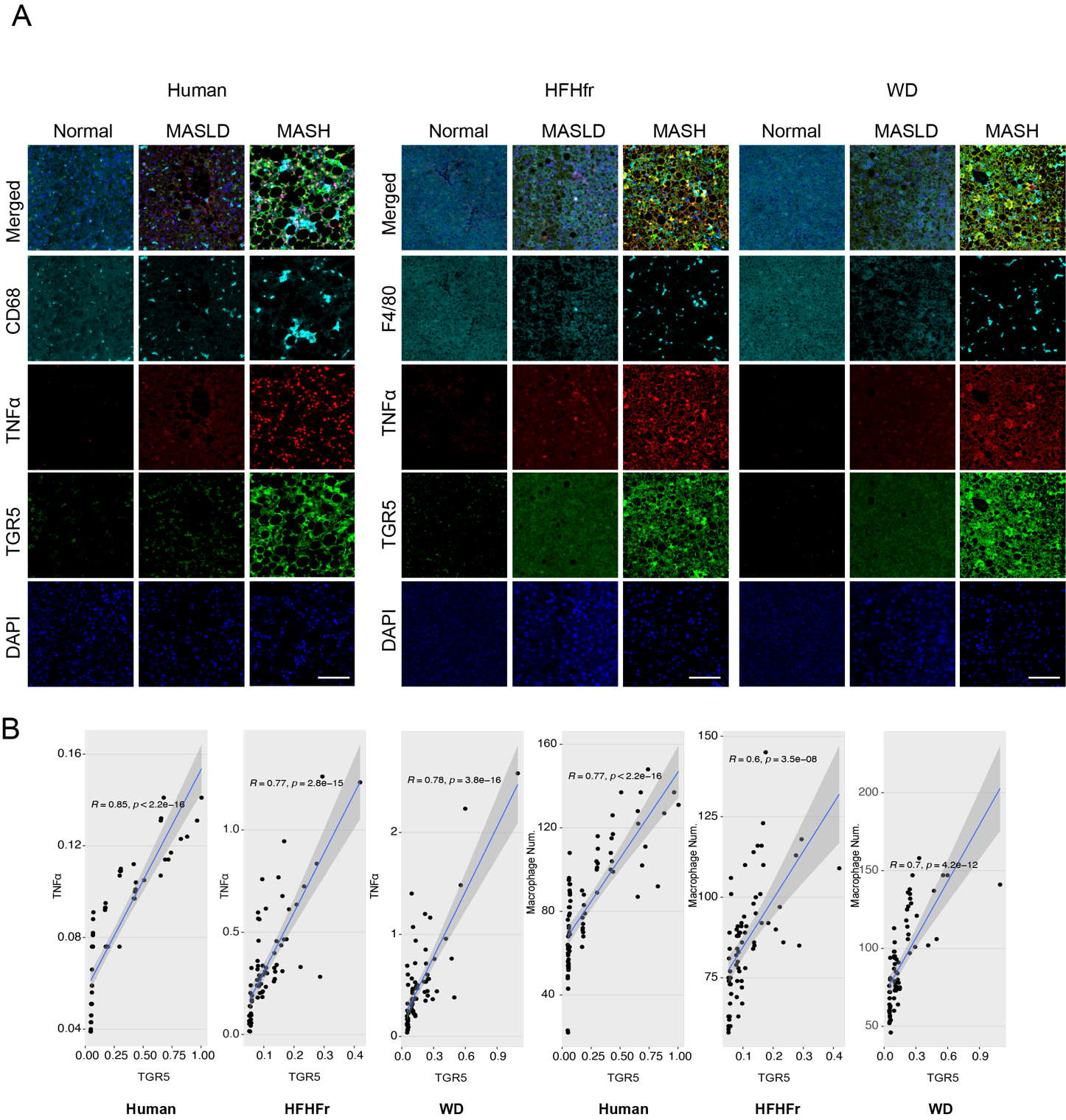


**Figure S2**

(A) mIHC staining of TGR5, CD68, F4/80 (CD68 for human and F4/80 for mice) and TNFα expression in human liver samples and mouse liver samples in two different models at different MASLD stages (Normal, MASLD, MASH).

(B) Correlations analysis of TGR5 with TNFα expression and macrophages numbers in human liver samples and mouse liver samples in two different models.

Data are presented as mean ± SD. All experiments were performed in triplicate.

**
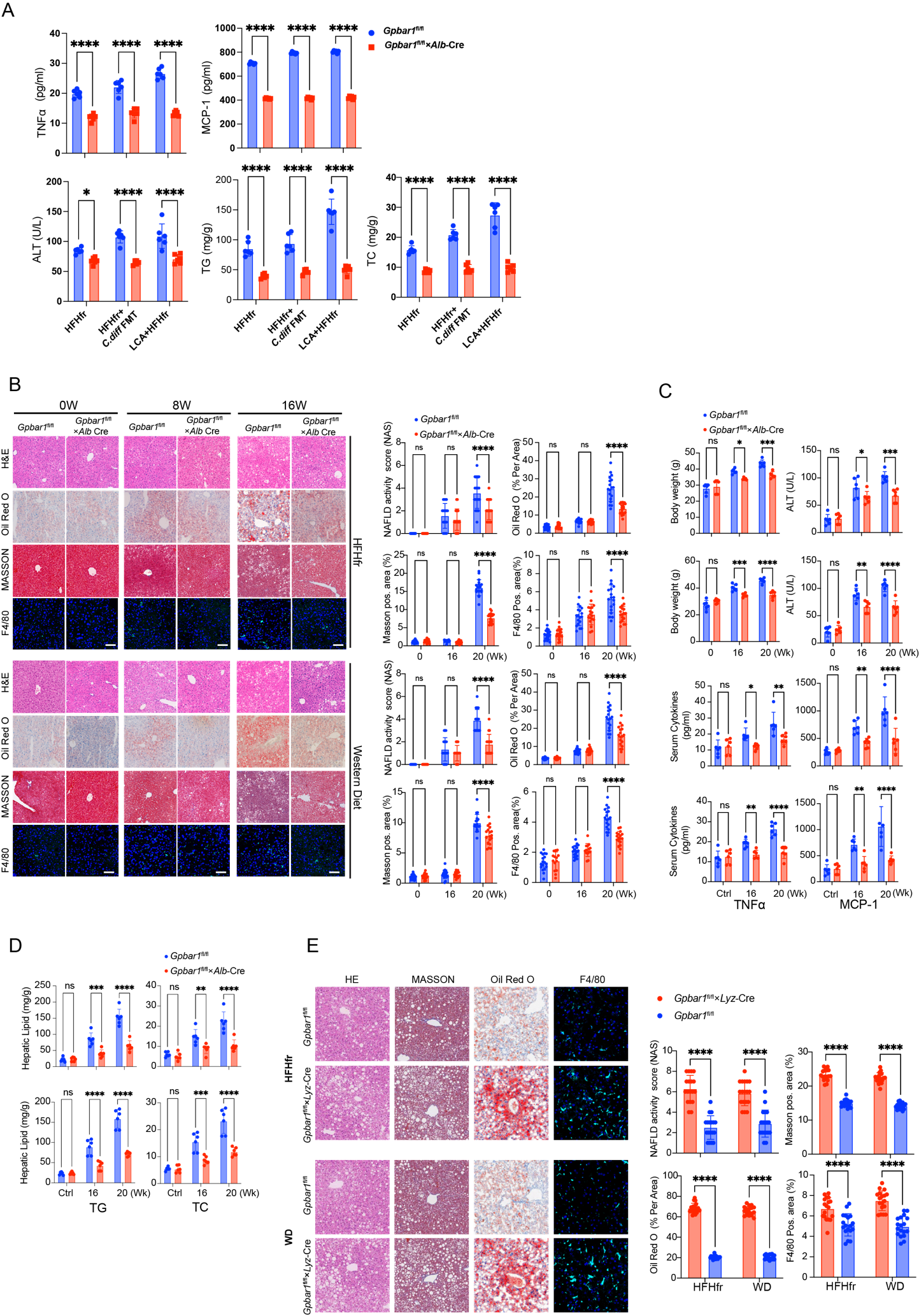
**

**Figure S3**

(A) Comparison of the TNFα, MCP-1, ALT levels, TG, and TC levels in *Gpbar1*^fl/fl^ and *Gpbar1*^fl/fl^×*Alb*-Cre mice at 16 weeks of the two conjugated LCA induced MASH model, each group n=6.

(B) Comparison of H&E, Oil Red O, Masson trichrome, F4/80 immunofluorescence staining, and NAS scores in *Gpbar1*^fl/fl^ and *Gpbar1*^fl/fl^×*Alb*-Cre mice at 0, 8, and 16 weeks of HFHfr and WD diet MASH model, each group n=18.

(C) and (D) Comparison of the body weight, TNFα, MCP-1, TG, and TC levels at different time points of HFHfr and WD diet MASH model, each group n=6. (A-D) Statistical significance was determined using on two-way ANOVA test with Šidák adjustment.

(E) Comparison of H&E, Oil Red O, Masson trichrome, F4/80 immunofluorescence staining, and NAS scores in *Gpbar1*^fl/fl^ and *Gpbar1*^fl/fl^×*Lyz*-Cre mice at 16 weeks of HFHfr and WD diet MASH model. Statistical significance was determined using one-way ANOVA test with Šidák adjustment, each group n=18.

Data are presented as mean ± SD. All experiments were performed in triplicate. **p*<0.05, ***p*<0.01, ****p*<0.0002, *****p*<0.0001, ns: *p*>0.05.


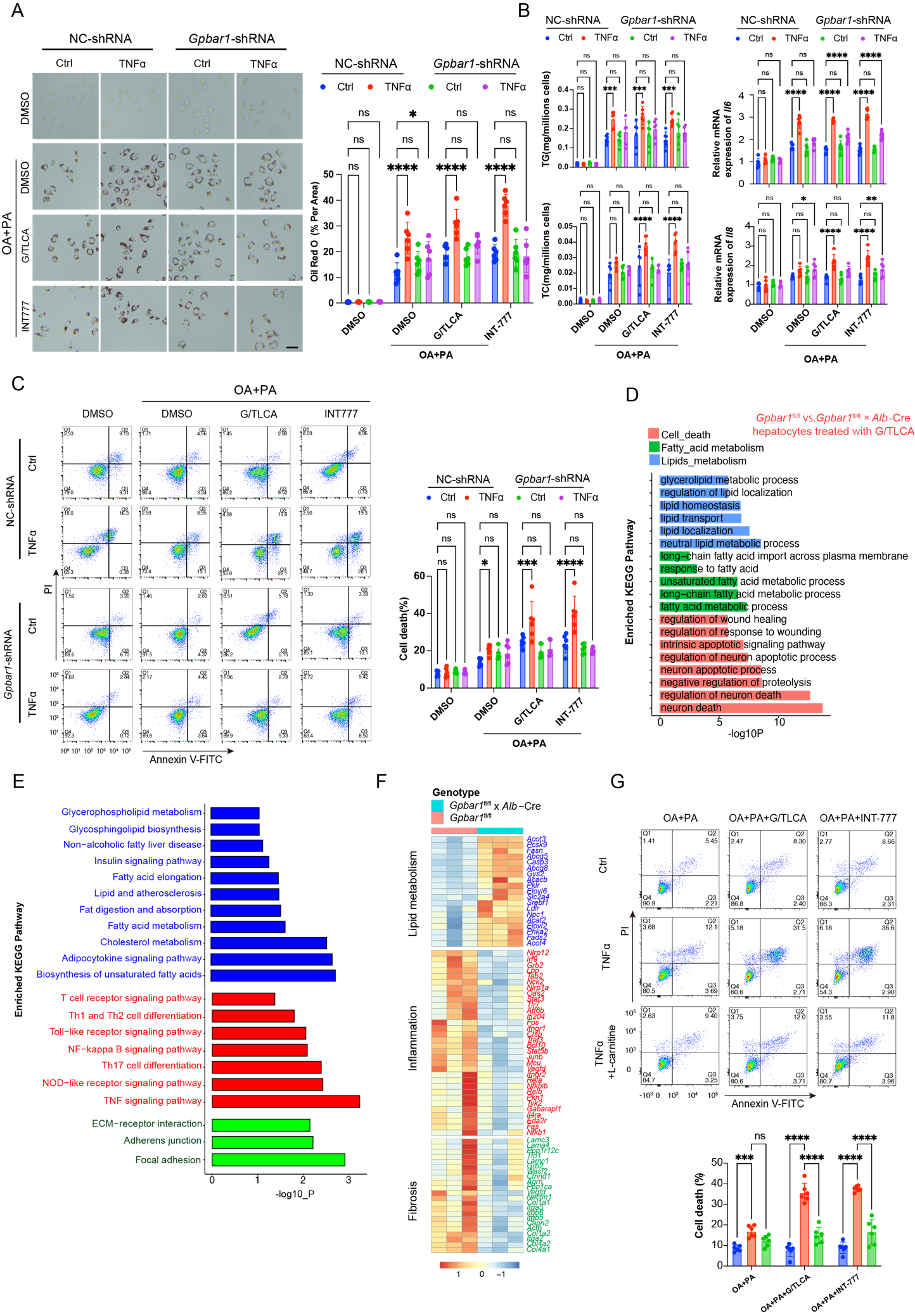


**Figure S4**

(A) and (B) Comparisons of Oil Red O, TG, TC levels, and the transcription levels of the *Il6* and *Il8* in AML12 after treatment as shown in the figure, each group n=6.

(C) Comparison of cell death rate in AML12 cells under different treatments shown in the figure, each group n=6. (A-C) Statistical significance was determined using two-way ANOVA test with Dunnett adjustment.

(D) GSEA analysis based on the KEGG dataset reflects the impact of 50μM G/TLCA on genes related to lipid metabolism, inflammation, and fibrosis in hepatocyte isolated from *Gpbar1*^fl/fl^ versus *Gpbar1*^fl/fl^×*Alb*-Cre mice.

(E) Enriched KEGG pathways associated with lipids metabolism, inflammation, and fibrosis are highlighted in liver samples from *Gpbar1*^fl/fl^ and *Gpbar1*^fl/fl^×*Alb*-Cre mice at 16 weeks of the LCA induced MASH model.

(F) Heatmap of differenteial gene expression involved in lipid metabolism, inflammation, and fibrosis in liver samples from *Gpbar1*^fl/fl^ and *Gpbar1*^fl/fl^×*Alb*-Cre mice at 16 weeks of the LCA induced MASH model.

(G) Comparison of cell death rate in AML12 cells under 50μM L-carnittine supplementation with different treatment. Statistical significance was determined using two-way ANOVA test with Šidák adjustment, each group n=6.

Data are presented as mean ± SD. All experiments were performed in triplicate. **p*<0.05, ****p*<0.0002, *****p*<0.0001, ns: *p*>0.05.


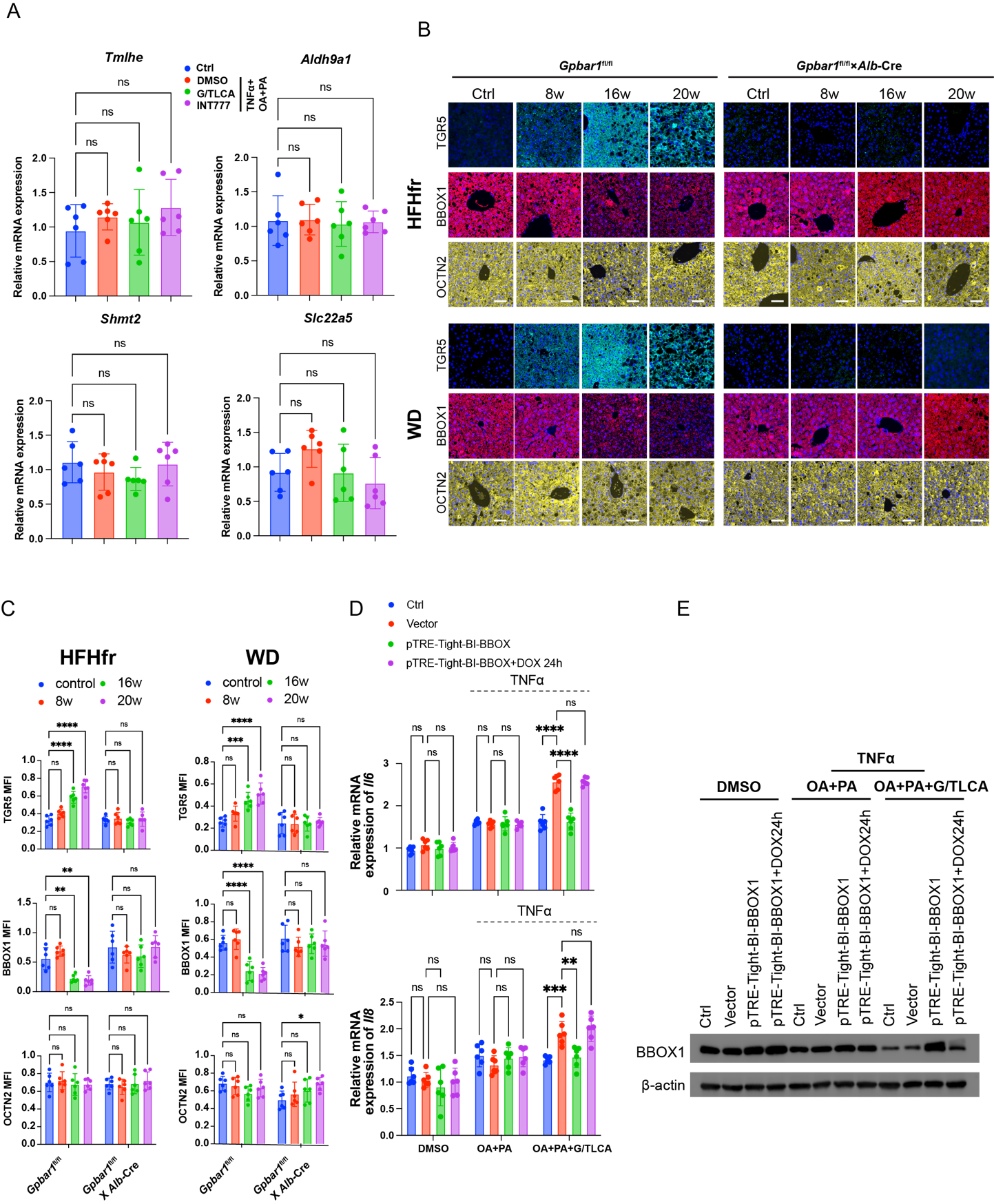


**Figure S5**

(A) Relative mRNA expression of *Tmlhe*, *Aldh9a1*, *Shmt2*, and *Slc22a5* in AML12 under different treatment shown in the figure. Statistical significance was determined using one-way ANOVA test with Dunnett adjustment, each group n=6.

(B) and (C) mIHC staining and MFI qualification of TGR5, BBOX1, OCTN2 expression in liver sections from *Gpbar1*^fl/fl^ and *Gpbar1*^fl/fl^×*Alb*-Cre mice treated with two MSAH models at different time point. Statistical significance was determined using two-way ANOVA test with Šidák adjustment, each group n=6.

(D) Comparison of the transcription of *Il6* and *Il8* in AML12 cells under different transfections and treatments. Statistical significance was determined using two-way ANOVA test with Dunnett adjustment, each group n=6.

(E) WB dynamically monitors the protein expression of BBOX1 in AML12 under different transfections and treatments, with β-actin as the internal control.

Data are presented as mean ± SD. All experiments were performed in triplicate. **p*<0.05, ***p*<0.01, ****p*<0.0002, *****p*<0.0001, ns: *p*>0.05.


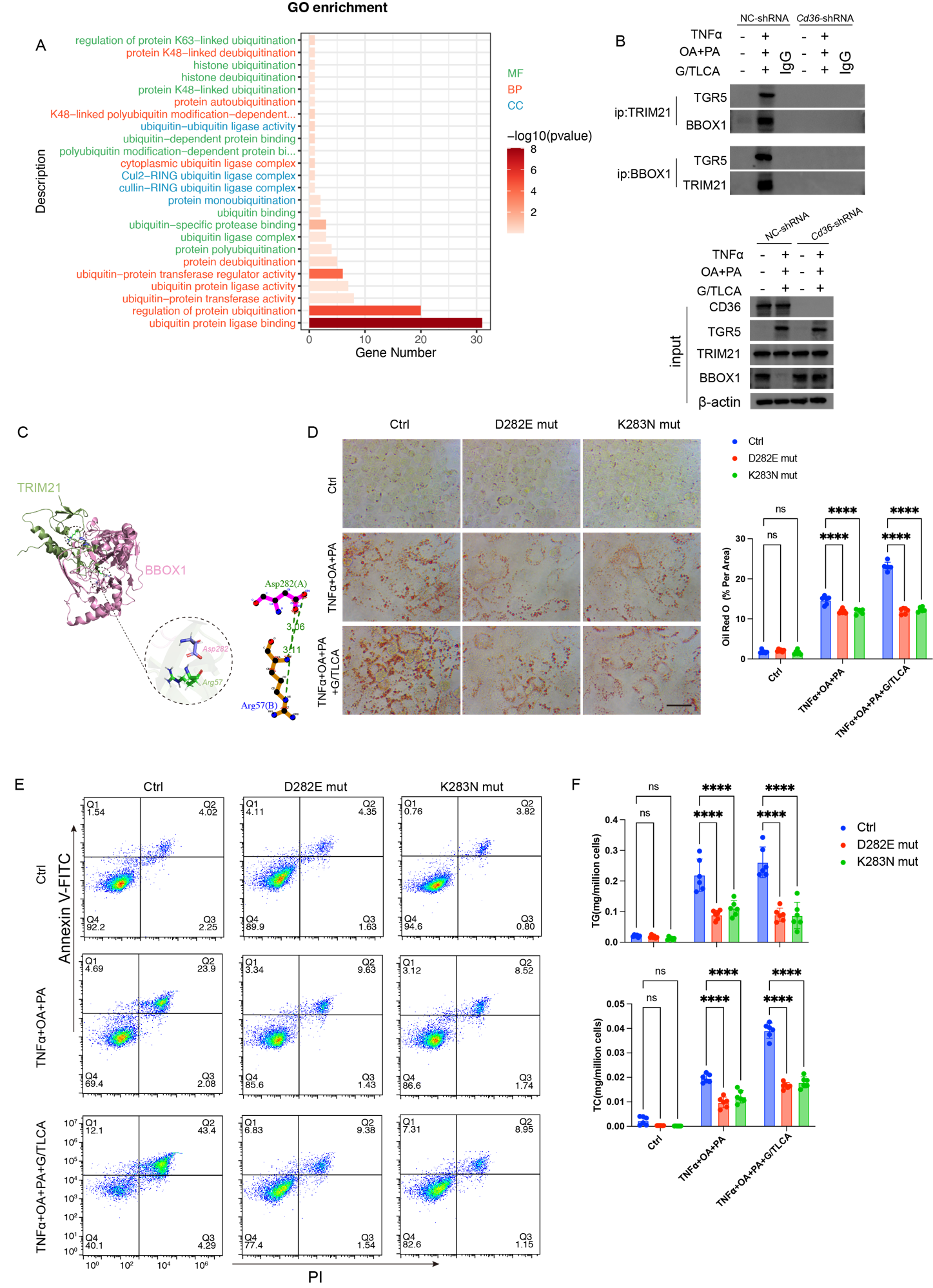


**Figure S6**

(A) GO analysis reflects the protein enriched by BBOX1 antibody with LC/MS.

(B) Co-IP and WB verification show the interactions between TRIM21, BBOX1 and TGR5 in AML12 and AML12 *Cd36*-shRNA under different treatment conditions. IgG serves as a negative control.

(C) Predicted docking sites and potential interaction interfaces between BBOX1 and TRIM21 receptors using Rosetta protein docking, visualized with PyMOL.

(D-F) Comparison of Oil Red O (D), cell death (E), TG and TC levels (F) of AML12 cells overexpressing wild-type and two mutant forms of BBOX1 treated under different treatments shown in the figure. Statistical significance was determined using two-way ANOVA test with Dunnett adjustment, each group n=6.

Data are presented as mean ± SD. All experiments were performed in triplicate. *****p*<0.0001.


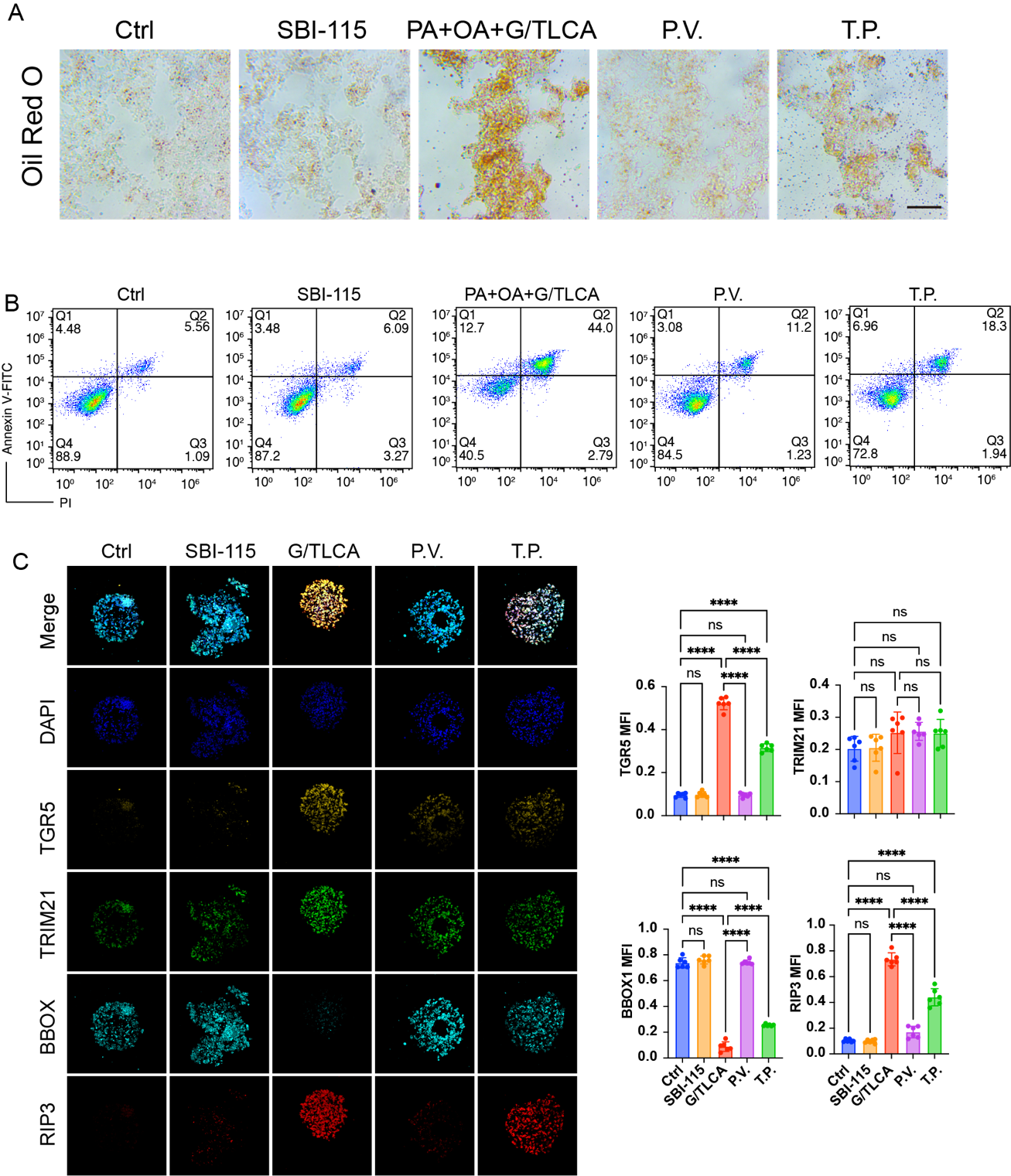


**Figure S7**

(A) and (B) Comparison of Oil Red O and cell death in the *in vitro* MASH model under different treatments.

(C) Representative images showing the expression of TGR5, TRIM21, BBOX1, and RIP3 under different treatments in the 3D *in vitro* model using mIHC staining. The model was constructed using the human THP-1 cell line and HepRG cells. Statistical significance was determined using on one-way ANOVA test with Šidák adjustment, each group n=6.


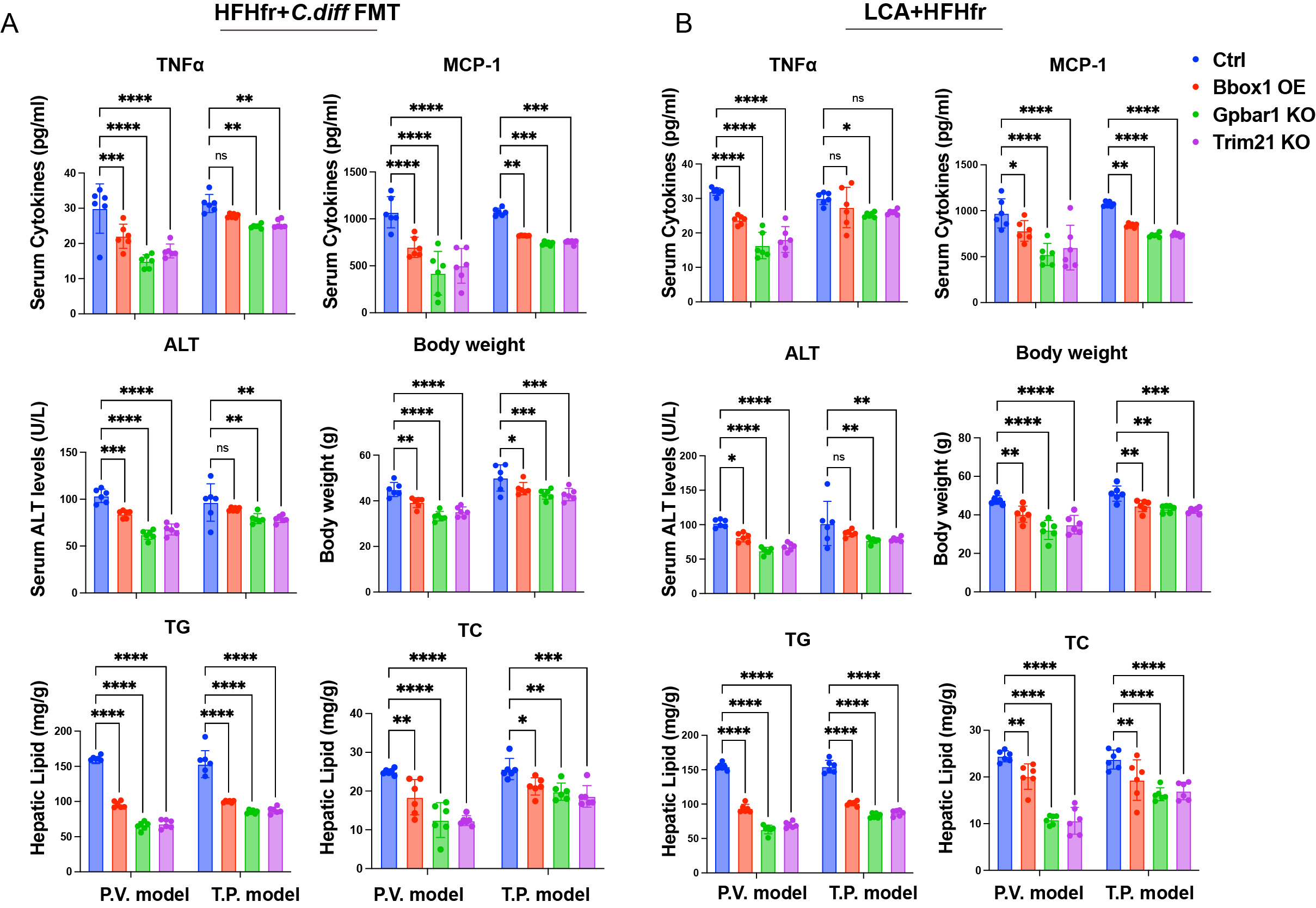


**Figure S8**

(A-B) Quantitative analysis of TNFα, MCP-1, ALT, TG and TC levels, and body weight of mice in the HFHFr+*C.diff* FMT mouse MASH model with prevention and therapeutic treatment. Statistical significance was determined using two-way ANOVA test with Dunnett adjustment, each group n=6.

Data are presented as mean ± SD. All experiments were performed in triplicate. **p*<0.05, ***p*<0.01, ****p*<0.0002, *****p*<0.0001, ns: *p*>0.05.

**Table S1 Primer sequences for qRT-PCR**

| **Gene Name** | **Species** | **Forward Sequence** | **Reverse Sequence** |
| --- | --- | --- | --- |
| *Shmt2* | Mouse | ACGCGTGTTGGAACTTGTCT | TCCAAGCCAATGTTGACTCCC T |
| *Bbox1* | Mouse | AGTGGTTCGCGTCAACTTCA | ATGAAGCAAGCGCCAGTTGT |
| *Actb* | Mouse | CTAGGCACCAGGGTGTGAT | CACGGTTGGCCTTAGGGTT |
| *Gpbar1* | Mouse | CCTGGCAAGCCTCATCGTC | AGCAGCCCGGCTAGTAGTAG |
| *Tmlhe* | Mouse | GAGAGGAAACATAGCTCAAGGTC | CCAGCATATTGCAGTTCCAAATG |
| *Aldh9a1* | Mouse | GGCCGAGTGATTGCCACTT | AGGCCACTTTTCTTACTCCAGA |
| *Slc22a5* | Mouse | ACTGTGCCAGGGGTGCTAT | GCAACTGAGGCTTCGTAGAAT |
| *Il6* | Mouse | TGGTCTTCTGGAGTACCATAGC | TGTGACTCCAGCTTATCTCTTGG |
| *Il8* | Mouse | CAAGGCTGGTCCATGCTCC | TGCTATCACTTCCTTTCTGTTGC |
| *SHMT2* | Human | GCCTCATTGACTACAACCAGCTG | ATGTCTGCCAGCAGGTGTGCTT |
| *ACTB* | Human | ATTCCTATGTGGGCGACGAG | AAGGTCTCAAACATGATCTGGGT |
| *GPBAR1* | Human | CCCAGGCTATCTTCCCAGC | GCCAGGACTGAGAGGAGCA |

**Reference**

[1] Liu, W. Y., Zheng, K. I., Pan, X. Y., et al., Effect of PNPLA3 polymorphism on diagnostic performance of various noninvasive markers for diagnosing and staging nonalcoholic fatty liver disease, 2020, J Gastroenterol Hepatol, 35, 1057, <https://doi.org/10.1111/jgh.14894>

[2] Liu, A. N., Xu, C. F., Liu, Y. R., et al., Secondary bile acids improve risk prediction for non-invasive identification of mild liver fibrosis in nonalcoholic fatty liver disease, 2023, Aliment Pharmacol Ther, 57, 872, <https://doi.org/10.1111/apt.17362>

[3] Zheng, C., Wang, L., Zou, T., et al., Ileitis promotes MASLD progression via bile acid modulation and enhanced TGR5 signaling in ileal CD8(+) T cells, 2024, J Hepatol, 80, 764, <https://doi.org/10.1016/j.jhep.2023.12.024>

[4] Li, W. C., Ralphs, K. L., & Tosh, D., Isolation and culture of adult mouse hepatocytes, 2010, Methods Mol Biol, 633, 185, <https://doi.org/10.1007/978-1-59745-019-5_13>
